# Changes in financial well-being and memory function and decline in middle-aged and older adults

**DOI:** 10.1093/aje/kwag054

**Published:** 2026-03-16

**Authors:** Katrina L Kezios, Jordan Vo, Zihan Chen, Sarah Weber, Allison E Aiello, Adina Zeki Al Hazzouri

**Affiliations:** Department of Epidemiology, Mailman School of Public Health, Columbia University, New York, NY, United States; Department of Epidemiology, Boston University School of Public Health, Boston, MA, United States; Weinberg College of Arts and Sciences, Northwestern University, Chicago, IL, United States; Department of Epidemiology, Mailman School of Public Health, Columbia University, New York, NY, United States; Department of Epidemiology, Boston University School of Public Health, Boston, MA, United States; Center for Trauma and Mental Health, Boston University School of Public Health, Boston, MA, United States; Department of Epidemiology, Mailman School of Public Health, Columbia University, New York, NY, United States; Robert N. Butler Columbia Aging Center, Mailman School of Public Health, Columbia University, New York, NY, United States; Department of Epidemiology, Mailman School of Public Health, Columbia University, New York, NY, United States

**Keywords:** financial well-being, economic insecurity, cognitive aging, memory decline, measurement

## Abstract

Many older adults experience financial insecurity. While prior studies link lower later-life socio economic status, financial stress, and financial shocks to worse cognitive outcomes, limited research has examined how dynamic changes in financial well-being—a multidimensional measure of financial circumstances—influence cognitive aging. Here, we examined associations between changes in financial well-being and memory outcomes among 7676 adults aged 50+ in the Health and Retirement Study (“HRS,” 2010-2020). We developed and validated an 8-item index of poor financial well-being using existing HRS survey items aligned with domains from the Consumer Financial Protection Bureau’s Financial Well-Being Scale. In confounder-adjusted linear mixed-effects models, we estimated the associations of average financial well-being and significant improvements or worsening in financial well-being over 4 years, with changes in memory *z* scores calculated biennially from 2016 to 2020. Each 1-point worsening in average financial well-being was associated with poorer memory function (*β* = -0.009 SD, 95% CI, -0.020 to 0.003) and accelerated decline (*β* = -0.007 SD/year, 95% CI, -0.010 to -0.003). Associations were largest for participants with significant worsening of financial well-being and for those aged ≥65 at baseline. Results were robust to sensitivity analyses addressing potential reverse causation and attrition. These findings suggest that midlife and later-life declines in financial well-being may contribute to accelerated cognitive aging.

## Introduction

Financial well-being is an emerging economic determinant of health that may be associated with cognitive aging.^1^^-^^3^ It reflects a sense of security and control over one’s finances and the ability to meet ongoing and future financial obligations, collectively providing the financial freedom and stability to enjoy life.^4^^,^^5^ Conversely, individuals with poor financial well-being often express financial dissatisfaction, have difficulty meeting basic needs, and feel insecure about their financial future, which negatively impacts overall well-being and life satisfaction.^6^^,^^7^ Prolonged experiences of poor financial well-being may lead to chronic stress, anxiety, and unhappiness, and worries about finances may overwhelm mental bandwidth, altogether contributing to negative cognitive outcomes as individuals age. Beyond these psychosocial pathways, poor financial well-being may also constrain investment in health-promoting resources such as nutritious food, healthcare, and social engagement, all of which are important for healthy cognitive aging.

Prior studies have not examined the cognitive consequences of poor financial well-being, although aspects of it—like economic hardship or ongoing financial strain^8^—have been shown to be risk factors for cognitive function and decline.^9^^-^^16^ Although conceptually similar to financial strain, poor financial well-being is a distinct construct that additionally incorporates domains related to one’s feelings of financial control, outlook, and satisfaction. When these elements are low or lacking, feelings of worry, anxiety, or unhappiness related to one’s financial situation and stress may be heightened, exacerbating their potential impact on cognitive outcomes. Yet, comprehensive measures of financial well-being from validated scales have only recently been developed and implemented in population-based research studies, making the literature on financial well-being and health fairly limited in general.^7^^,^^17^^-^^25^ Still, among existing studies, poor financial well-being has been associated with heightened stress,^20^^,^^21^ anxiety,^21^^,^^22^ and depression,^23^^-^^25^ and these outcomes may increase the risk of cognitive decline and dementia.^26^

To better understand how financial well-being and changes in financial well-being in midlife and older adulthood impact cognitive aging, we use data from the Health and Retirement Study (HRS) to construct an index of poor financial well-being from existing survey items and examine its longitudinal relationship with changes in memory function. Our index was designed to capture poor financial well-being as a multidimensional exposure encompassing both a lack of psychosocial resources (eg, perceived financial dissatisfaction and strain) and material constraints (eg, difficulty meeting basic needs, low income). We validated our index against the Consumer Financial Protection Bureau’s Financial Well-Being (CFPB-FWB) Scale,^5^ which was first introduced in the HRS in 2020.

## Methods

### Sample

The HRS is a national longitudinal study of US adults aged 51 and older at recruitment (and their spouses regardless of age).^27^ The original cohort was recruited in 1992, with new “refresher” cohorts added every 6 years; after recruitment, participants are re-surveyed biennially. Each biennial survey collects detailed information on participants’ household finances (eg, income from multiple sources). In alternating 4 year cycles, 2 rotating subsamples complete a leave-behind self-administered psychosocial and lifestyle questionnaire (SAQ), which asks participants questions about their financial circumstances (eg, financial satisfaction, financial control). Subsample A completed the SAQ in 2006/2010/2014/etc., and subsample B in 2008/2012/2016/etc.^28^

Our sample for this analysis included participants aged >50 who were eligible for and completed the SAQ in either 2010 (exposure baseline) and 2014 (exposure follow-up) or 2012 (exposure baseline) and 2016 (exposure follow-up). Before accounting for missing data and other considerations, the combined sample size was *N* = 10 277. As described in detail below (and illustrated in Figure S1), we then restricted our sample to participants with complete data on financial well-being at both exposure baseline and follow-up (*N* = 9188), non-missing covariate data (*N* = 8920), and at least 1 cognitive outcome recorded between 2016 and 2020, making the final analytic sample size *N* = 7676.

### Exposure

#### Operationalizing financial well-being

We constructed an index of poor financial well-being at exposure baseline and 4 year follow-up from 8 existing HRS survey items reflecting participants’ household finances and perceived financial circumstances. These items were chosen (1) to align with other conceptualizations/assessments of financial well-being in the health sciences literature (eg, income, wealth, financial strain); (2) to capture domains represented in the CFPB-FWB Scale^5^^,^^29^; and (3) because they were measured in multiple HRS surveys over time. The original question wording, answer choices, and variable names/data sources for each indicator are presented in Appendix S1 and Tables S1A and B.

The 8 items that were recoded into binary indicators included: *low financial control* (score of 0-4 vs 5-10, where 0 = “no control at all” and 10 = “total control”), *financial dissatisfaction* (not very/not at all vs somewhat/very/completely satisfied), *difficulty paying bills* (very/completely vs somewhat/not very/not at all difficult), *ongoing financial strain* (yes and very/somewhat upsetting vs yes but not upsetting/didn’t happen), *taking less medication because of money* (yes vs no), *low income* (net family income—equivalized for family size^30^ and scaled for a family of 4—that is <125% the poverty line for a family of 4 in a given year), *no housing wealth* (not owning home/a net value of primary residence ≤ 0), and *low non-housing wealth* (total non-housing wealth—eg, savings, stocks—in bottom quartile of the sample at a given time point). Each binary indicator was coded 1 vs 0, with 1 representing “worse” financial status. We summed across indicators and summary scores ranged from 0 to 8, with higher scores indicating worse financial well-being.

We operationalized financial well-being over time in 3 ways: average financial well-being, which averaged index scores at exposure baseline and follow-up; significant improvement in financial well-being, defined as a ≥2-point drop in score from baseline to follow-up (vs all else); and *significant worsening of financial well-being,* defined as a ≥2-point increase in score from baseline to follow-up (vs all else). The ≥2-point threshold was chosen to capture meaningful change in financial well-being while minimizing misclassification due to short-term fluctuation or measurement error in individual items. Because summary scores range from 0 to 8, a 2-point change (vs 1-point) is more likely to reflect shifts across multiple domains (eg, income and perceived financial strain), rather than a change in a single indicator. This cut point (vs more stringent thresholds) also ensured sufficient sample sizes in the improvement and worsening groups for stable estimation.

#### Validating our financial well-being index

In 2020, the HRS included the CFPB-FWB Scale in the SAQ in addition to its typical collection of the financial indicators considered herein. Using the 2020 HRS survey as a validation sample, we computed and compared participants’ scores on both our index and the CFPB-FWB Scale. Specifically, we assessed correlations between index scores and CFPB-FWB scores (convergent validity) and financial literacy scores (discriminant validity). We also evaluated the internal consistency of our index with Cronbach’s *α*. Finally, we compared the magnitude of associations between both financial well-being measures and 4 validation outcomes (fair/poor self-rated health, receiving Medicaid, receiving income from government assistance, and food insecurity). Complete details of our validation approach and methods are presented in Appendix S1.

### Outcomes

Our outcome of interest was a previously validated composite memory score predicted from an algorithm that considered both direct memory assessments (immediate and delayed 10-word recall tasks) and, for those too cognitively impaired to complete direct assessments, proxy assessments of memory function (proxy’s assessment of participants’ memory performance on a 5-point Likert scale and the 16-item Jorm Informant Questionnaire for Cognitive Decline).^31^ To align temporally with our exposure periods, we assessed memory decline from 2016 to 2020 (3 time points of measurement), with scores standardized to the sample distribution in 2016.

### Confounders

Confounders were identified *a priori* through literature review as potential common causes of baseline financial well-being and/or changes in financial well-being and cognitive outcomes. All confounders were measured at exposure baseline (ie, at the start of the 4 year exposure window) and prior to outcome follow-up. Confounders included: age (continuous, years), race (Black or other race, White^*^), sex/gender (female, male^*^), educational attainment (years, continuous), being born in the Southern United States (born in South Atlantic/East South Central/West South Central Census regions vs New England/Mid-Atlantic/East North Central/West North Central/Mountain/Pacific^*^), parental education (highest of mother or father’s categorized as <8, 8-12^*^, >12 years, unknown), marital status (married/partnered^*^, separated/divorced/widowed, never married), having employer-provided health insurance (no, yes^*^), occupational skill level/working status (higher skill^*^, lower skill, not working, unknown), wealth (sum of all household sources of assets minus debt and equivalized for household size, continuous), income (sum of all household sources income and equivalized for household size, continuous), drinking status (ever drinks any alcohol beverages, never drinks^*^), smoking status (current, former/never^*^), body mass index (BMI) (kg/m^2^, continuous), number of doctor-diagnosed health conditions (self-reported, continuous), and elevated depressive symptoms (scores on the 8-item Center for Epidemiological Studies of ≥4, <4^*^). The reference group for analyses is noted with an asterisk^*^.

### Modifiers

We examined modification by age at exposure baseline (51-64 vs ≥65 years) to explore whether financial well-being and changes in financial well-being impact cognitive outcomes differently at different life stages.

### Statistical analysis

We used linear mixed-effects models (LMMs) to estimate the effect of each exposure on 4 year memory decline.^32^ LMMs used time on study as the timescale, adjusted for baseline age, and included a random intercept and random slope with a linear time term, given the short period of follow-up.

We used a sequential adjustment strategy to evaluate how associations changed with adjustment for progressively broader sets of covariates. Model 1 was minimally adjusted and included only age (centered at 65 years), age-squared, and their interactions with time. Model 2 was additionally adjusted for time-invariant early-life and life-course sociodemographic confounders: race, sex/gender and its interaction with time, years of education (centered at 12) and its interaction with time, Southern birthplace, and parental education. Model 3 was additionally adjusted for baseline socioeconomic and occupation-related confounders: marital status, having employer-provided health insurance, occupational skill level/working status, total household wealth, and total household income (log-transformed after adding a small correction factor of 0.001). Including continuous income and wealth in this model helped account for residual variation in objective financial resources at baseline beyond the binarized indicators used in the financial well-being index. Finally, model 4 was additionally adjusted for baseline measures of health status and behaviors: alcohol drinking status, smoking status, BMI (mean-centered), number of chronic health conditions, and depressive symptomatology. Although measured prior to observed changes in financial well-being and outcome follow-up, their concurrent measurement with exposure baseline introduces ambiguity regarding whether these variables act as confounders or mediators. We therefore present models including these variables separately.

Covariate–by-time interactions were tested for all confounders in model 4 to allow confounders to vary with memory decline over time. Only interactions with education and both age terms were statistically significant; we also retained the sex/gender–by-time interaction based on theoretical considerations. In a model including interactions with time for all confounders, results were similar to the main models, but estimates were less precise due to increased model complexity. We did not adjust for practice effects as nearly all participants had completed at least 1 memory assessment prior to 2016.

To explore effect modification by age at exposure baseline, we ran models for each exposure in the overall sample and within age strata (51-64 vs ≥65). We also formally tested for effect modification of both baseline memory function and memory decline by including 3 way interaction terms between financial well-being exposure, age at exposure baseline, and time in the LMMs (defining statistical significance as *P* < .10). We visualized findings from fully adjusted models by plotting predicted memory score trajectories over the 4 years of outcome follow-up. Predicted scores at each time point were generated for reference-level characteristics (defined above).

All analyses were conducted using R version 4.5.0 (R Core Team, 2021).

### Sensitivity analysis

Because some time-varying variables may help explain changes in financial well-being, we extended model 4 to adjust for experiencing a marriage ending, becoming unemployed, experiencing significant weight loss (>1 SD decrease in BMI), and reporting a new health condition from exposure baseline to follow-up and each of their interactions with time.

To address potential reverse causation—that is, that changes in some financial indicators may be early warning signs of dementia (eg, difficulty paying bills, accumulating debts/decreasing assets)^33^^,^^34^—we excluded participants with baseline memory scores ≥ 2 SD below the sample mean and reran primary analyses. In a separate sensitivity analysis, we used changes in 2016-2020 memory *z* scores from baseline (2010/2012) as the outcome, to better account for pre-existing cognitive differences that could influence both financial well-being and memory decline.^35^

Finally, to assess potential bias from selective attrition, we examined changes in sample composition across sample restrictions (Figure S1) and applied stabilized inverse probability of censoring weights (IPW)—estimated from baseline sociodemographic, financial, health, and cognitive characteristics and defined as the inverse probability of being a complete case versus censored among the original sample (*N* = 10 277)—to reweight estimates to the eligible population. Weighted LMMs were fit using the WeMix package in R,^36^ which accommodates IPW in multilevel longitudinal data, with stabilized participant-level weights and observation-level weights fixed at 1.

## Results

### Sample characteristics

Table 1 presents the sample characteristics, overall and by age group. At exposure baseline, participants averaged 66 years of age and 13.5 years of education; ~ 60% identified as female; ~20% identified as Black or another race; and ~70% were married or partnered. Median household income at baseline ($49 200, in 2010 dollars^37^) closely matched the 2010 US median ($49 280). The average financial well-being score over 4 years was 1.33 (SD = 1.64), with 11.5% of the sample experiencing a significant improvement in scores and 7.3% a significant worsening.

**Table 1:** Baseline characteristics of the analytic sample of US Health and Retirement Study participants (2010-2020), overall and within age strata.

		**Age group**	
**Baseline characteristics** ^**a**^	**Overall**	**51-64**	**≥65**
	**(*N* = 7676)**	**(*N* = 3608)**	**(*N* = 4068)**
Age at baseline (years)			
Mean (SD)	66.2 (9.50)	57.7 (3.82)	73.8 (5.94)
Median [Q1-Q3]	66.0 [58.0-73.0]	58.0 [54.0-61.0]	73.0 [69.0-78.0]
Race^b^			
Black or other	1499 (19.5%)	971 (26.9%)	528 (13.0%)
White	6177 (80.5%)	2637 (73.1%)	3540 (87.0%)
Gender^c^			
Female	4592 (59.8%)	2183 (60.5%)	2409 (59.2%)
Male	3084 (40.2%)	1425 (39.5%)	1659 (40.8%)
Years of education			
Mean (SD)	13.5 (2.45)	13.8 (2.33)	13.2 (2.52)
Median [Q1-Q3]	13.0 [12.0-16.0]	14.0 [12.0-16.0]	12.0 [12.0-16.0]
Born in a Southern US state			
Yes	2327 (30.3%)	1042 (28.9%)	1285 (31.6%)
No	5349 (69.7%)	2566 (71.1%)	2783 (68.4%)
Parental years of education^d^			
<8 years	752 (9.8%)	241 (6.7%)	511 (12.6%)
8-12 years	4670 (60.8%)	2106 (58.4%)	2564 (63.0%)
>12 years	1914 (24.9%)	1097 (30.4%)	817 (20.1%)
Unknown	340 (4.4%)	164 (4.5%)	176 (4.3%)
Marital status			
Married/partnered	5234 (68.2%)	2548 (70.6%)	2686 (66.0%)
Separated/divorced/widowed	2098 (27.3%)	809 (22.4%)	1289 (31.7%)
Never married	344 (4.5%)	251 (7.0%)	93 (2.3%)
Has employer-provided health insurance			
Yes	3798 (49.5%)	2435 (67.5%)	1363 (33.5%)
No	3878 (50.5%)	1173 (32.5%)	2705 (66.5%)
Occupational skill level			
Higher skill	1269 (16.5%)	968 (26.8%)	301 (7.4%)
Lower skill	1636 (21.3%)	1125 (31.2%)	511 (12.6%)
Not working	4530 (59.0%)	1305 (36.2%)	3225 (79.3%)
Unknown	241 (3.1%)	210 (5.8%)	31 (0.8%)
Wealth at baseline (in 2010 dollars)^e^			
Mean (SD)	483 000 (848 000)	406 000 (860 000)	552 000 (832 000)
Median [Q1-Q3]	214 000 [54 300-567 000]	146 000 [20 400-456 000]	281 000 [96 800-688 000]
Income at baseline (in 2010 dollars)^f^			
Mean (SD)	73 800 (93 100)	88 100 (103 000)	61 100 (81 400)
Median [Q1-Q3]	49 200 [25 900-88 300]	62 500 [29 700-111 000]	40 700 [24 500-70 200]
Drinking status			
Ever drinks alcohol	4611 (60.1%)	2374 (65.8%)	2237 (55.0%)
Never drinks alcohol	3065 (39.9%)	1234 (34.2%)	1831 (45.0%)
Smoking status			
Currently smokes cigarettes	936 (12.2%)	656 (18.2%)	280 (6.9%)
Never/formerly smoked cigarettes	6740 (87.8%)	2952 (81.8%)	3788 (93.1%)
Body mass index			
Mean (SD)	28.9 (6.07)	29.5 (6.46)	28.4 (5.64)
Median [Q1-Q3]	27.9 [24.7-32.1]	28.3 [24.9-32.9]	27.5 [24.4-31.3]
No. of doctor-diagnosed health conditions			
Mean (SD)	1.94 (1.38)	1.57 (1.35)	2.28 (1.32)
Median [Q1-Q3]	2.00 [1.00-3.00]	1.00 [1.00-2.00]	2.00 [1.00-3.00]
Depressive symptoms (CESD-8 score ≥ 4)			
Yes	840 (10.9%)	496 (13.7%)	344 (8.5%)
No	6836 (89.1%)	3112 (86.3%)	3724 (91.5%)
Marriage/partnership ended			
Yes	479 (6.2%)	172 (4.8%)	307 (7.5%)
No	7197 (93.8%)	3436 (95.2%)	3761 (92.5%)
Became unemployed			
Yes	173 (2.3%)	110 (3.0%)	63 (1.5%)
No	7503 (97.7%)	3498 (97.0%)	4005 (98.5%)
Experienced significant weight loss			
Yes	772 (10.1%)	332 (9.2%)	440 (10.8%)
No	6864 (89.4%)	3256 (90.2%)	3608 (88.7%)
Missing	40 (0.5%)	20 (0.6%)	20 (0.5%)
Experienced ≥ 1 newly diagnosed health condition			
Yes	2265 (29.5%)	1046 (29.0%)	1219 (30.0%)
No	5411 (70.5%)	2562 (71.0%)	2849 (70.0%)
Average FWB score over time			
Mean (SD)	1.33 (1.64)	1.69 (1.82)	1.01 (1.38)
Median [Q1-Q3]	0.50 [0-2.00]	1.00 [0-3.00]	0.50 [0-1.50]
FWB significantly improved over time			
Yes	879 (11.5%)	601 (16.7%)	278 (6.8%)
No	6797 (88.5%)	3007 (83.3%)	3790 (93.2%)
FWB significantly worsened over time			
Yes	559 (7.3%)	275 (7.6%)	284 (7.0%)
No	7117 (92.7%)	3333 (92.4%)	3784 (93.0%)
Composite memory *z* score at baseline			
Mean (SD)	0.36 (0.73)	0.72 (0.45)	0.037 (0.78)
Median [Q1-Q3]	0.50 [-0.0047-0.85]	0.77 [0.48-1.00]	0.17 [-0.37-0.50]
Missing	22 (0.3%)	4 (0.1%)	18 (0.4%)

Abbreviations: CESD-8, 8-item version of the Center for Epidemiologic Studies Depression Scale; FWB, financial well-being; No., number.

^a^Baseline is the year of first financial well-being measure, either 2010 or 2012 in the combined sample.

^b^Self-reported by participants and collapsed into categories of Black, White, or another racial group.

^c^Self-reported by participants as male or female (no distinction in surveys between sex/gender).

d Highest of either parent’s reported years of education.

e Sum of all sources of household assets minus debts reported in 2010 dollars.

f Sum of all sources of household income reported in 2010 dollars.

Compared with participants aged 51-64 (*N* = 3608), those ≥65 (*N* = 4068) were more likely to be White, separated/divorced/widowed, and not working (overwhelmingly due to retirement) at exposure baseline; they also had higher wealth but lower average financial well-being, income, and memory *z* scores (Table 1). Median incomes for the 51-64 ($62 500) and ≥65 ($40 700) age groups were slightly higher than corresponding age group-specific 2010 US medians (45-54 years: $62 340; 55-64 years: $56 470; ≥65 years: $31 460).^37^ Participants aged 51-64 were more likely to experience significant improvements in financial well-being over time. Across both age groups, participants experiencing a significant change in financial well-being, regardless of direction, were more often female, Black or another race, were separated/divorced/widowed, and had lower socioeconomic status and more depressive symptoms at baseline (Appendix S2, Tables S2A and B).

Participants in the final analytic sample, compared with the starting sample of *N* = 10 277, were only modestly healthier and more socioeconomically advantaged, suggesting that selection processes were unlikely to strongly bias our findings (Tables S2C-E).

### Exposure validation

Our financial well-being index showed good internal consistency (Cronbach’s *α* = 0.702). It was well correlated in the expected direction with the CFPB-FWB Scale (${\rho}_{\mathrm{Spearman}}=$-0.65, 95% CI, -0.67 to -0.63), whereas its correlation with financial literacy was noticeably lower (${\rho}_{\mathrm{Spearman}}$= -0.17, 95% CI, -0.27 to -0.06), providing evidence of good convergent and discriminant validity, respectively. Detailed validation results are presented in Appendix S3, Tables S2A-C, and Figure S2.

### Regression results

**Figure 1:**
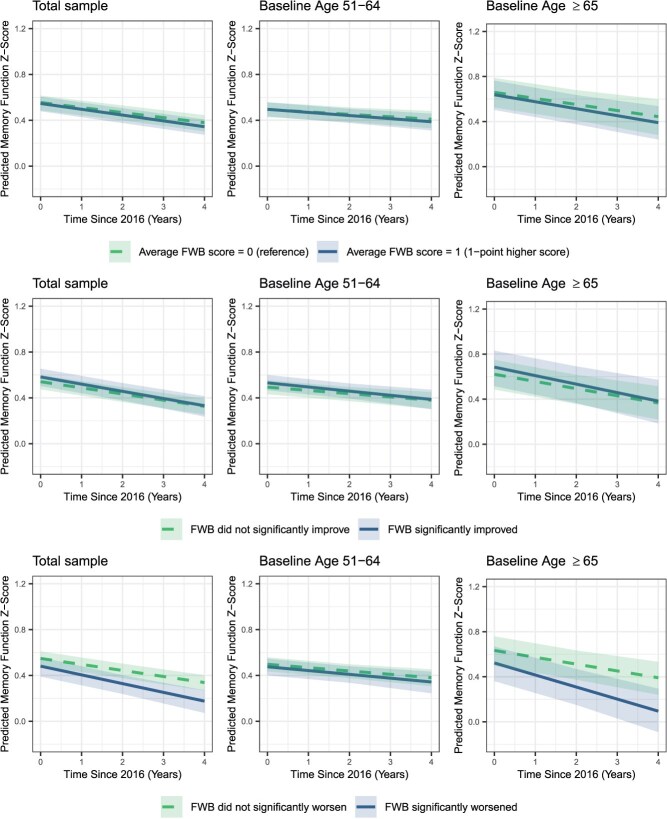
Predicted memory decline trajectories for each financial well-being exposure group in the total sample and within age strata. The top panel shows predicted memory trajectories from fully adjusted models at 2 illustrative values of average financial well-being (FWB = 0 [reference] vs FWB = 1), representing a 1-point difference in the continuous FWB score (with higher scores indicating worse financial well-being). The middle panel contrasts predicted memory decline trajectories for participants whose financial well-being significantly improved over time vs not. The bottom panel contrasts predicted memory decline trajectories for participants whose financial well-being significantly worsened over time vs not. Predicted scores at each time point were generated for reference-level characteristics. Abbreviation: FWB, financial well-being.

Overall, in fully adjusted models, each 1-point increase (worsening) in average financial well-being was associated with lower memory function in 2016 ($\beta$= -0.009 SD, 95% CI, -0.020 to 0.003) and accelerated memory decline from 2016 to 2020 ($\beta$= -0.007 SDs per year, 95% CI, -0.010 to -0.003) (Figure 1, row 1). Relative to the average annual rate of memory decline (the time coefficient in the model), this corresponds to approximately 2 additional months of memory aging per year. Associations were especially large for participants whose financial well-being score significantly worsened over 4 years (memory function: $\beta$= -0.067 SD, 95% CI, -0.122 to -0.013; memory decline: $\beta$= -0.024 SD, 95% CI, -0.043 to -0.004; Figure 1, row 3), whereas significant improvements in financial well-being were unassociated with memory function or decline (Figure 1, row 2). For participants with significant worsening in financial well-being, the acceleration in memory decline was equivalent to approximately 5 additional months of memory aging per year.

In general, associations were larger (ie, worse memory function and faster decline), although less precisely estimated, for participants aged ≥65 (Figure 1, column 3) vs 51-64 (Figure 1, column 2), especially for the worsening financial well-being exposure. Age-group differences in baseline memory function were statistically significant for average financial well-being and worsening financial well-being, while differences in rates of decline were statistically significant only for worsening financial well-being. All estimates corresponding to Figure 1 are shown in (Appendix S4, Table S4A).

Appendix S4 contains results from sensitivity analyses (Tables S4B-E). Conclusions were generally consistent (although slightly attenuated, mostly for participants aged ≥65) when *N* = 350 participants with low memory scores at exposure baseline were removed (Table S4B). Findings were also largely consistent when the outcome accounted for differences in memory scores at exposure baseline; most notably, there was some attenuation in estimates of memory function (especially for the worsening financial well-being exposure), but not memory decline across groups (Table S4C). Adjustment for variables whose change over time could also influence changes in financial well-being (eg, becoming unemployed) also resulted in estimates similar to those from primary analyses (Table S4D, model 4s). Finally, weighted estimates were similar in direction and just slightly larger in magnitude than complete-case results, suggesting that estimates from our primary analyses are robust and potentially conservative (Table S4E).

## Discussion

Overall, worse average financial well-being and declines in financial well-being over a 4 year period in midlife and older age were associated with lower memory function scores and faster memory decline over the next 4 years. The strongest associations were observed for individuals aged ≥65 at baseline and were robust to strategies to deal with potential reverse causation or selective attrition.

Our findings contribute to growing evidence suggesting that recent financial hardship or significant declines in financial status during midlife and older age could negatively impact brain health. For example, 2 recent studies reported that middle-aged and/or older US adults experiencing wealth shocks showed worse cognitive function,^38^ accelerated cognitive decline,^13^ and higher dementia risk.^13^ In 2 other studies, midlife financial stress—defined as financial dissatisfaction and a change in one’s financial situation—was associated with poorer later-life cognitive function,^10^ while improvements in financial stress were associated with reduced dementia risk.^39^ We similarly observed that improved financial well-being was associated with higher memory function in both age groups (although not statistically significantly for those aged ≥65). Additionally, consistent with our age-stratified findings, Kiely et al. found that experiencing a recent financial crisis was associated with negative changes in cognitive test performance, particularly for memory outcomes among those aged 60+ years in their sample.^40^ Because older adults typically rely on reduced fixed incomes (eg, social security, pensions, savings), changes to their financial well-being may be more challenging to navigate, with fewer options for recovery^41^^,^^42^; this could worsen cognitive health by increasing stress or reducing access to other health resources that are cognitively protective. While midlife changes in financial well-being can also profoundly affect financial security and retirement plans, middle-aged adults may have more options and flexibility to buffer financial setbacks—such as the ability to continue working—and benefit from having more time to recover financially.^43^^,^^44^ Because of this reduced capacity for financial recovery in later life, financial assistance may be especially important for protecting cognitive health among older adults. Supporting this, evidence from randomized and quasi-experimental evaluations of cash transfers and pension policies suggests that income supports in later life may slow cognitive decline and reduce dementia risk.^45^^-^^47^

Although our findings align with prior work, a potential alternative explanation for the strong associations observed among participants aged ≥65 is reverse causation. There is a complex and potentially bidirectional relationship between financial well-being and cognitive health in older adults. Financial stress and hardship can negatively impact cognitive function, while cognitive decline can in turn threaten financial stability. Indeed, negative changes in financial decision-making (eg, difficulty managing bills) are among the early functional changes in individuals with incipient dementia.^33^ However, our findings were robust to multiple strategies addressing differences in cognitive function already apparent at baseline, including removing people with low memory scores and repeating analyses with the outcome operationalized as change from baseline. Further, we observed associations with younger participants in the sample as well (those aged 50-64), even with the removal of participants with poor baseline memory function. This suggests our findings are likely not entirely due to potential reverse causation.

Other limitations of our study include potential attrition bias and residual confounding. While our outcome measure included responses by proxy, which helps limit potential attrition bias dependent on the outcome, it does not eliminate this threat. If participants with no direct cognitive assessments or proxy reports dropped out of the sample for reasons related to poor financial well-being and cognitive impairment, our observed findings may be underestimated; results from our sensitivity analyses suggest that any bias from selective attrition is likely modest and that our estimates are conservative. Additionally, health changes that affect both financial well-being and cognitive function could bias results, especially among participants aged ≥65 at baseline, who are more likely to experience health shocks that cause financial strain.^48^ However, we controlled for doctor-diagnosed health conditions and BMI at baseline, as well as changes in these covariates over the exposure period in a sensitivity analysis, and findings remained strong. Further, because baseline health status and behaviors were measured concurrently with baseline financial well-being, we cannot definitively distinguish confounding from mediation, and estimates from fully adjusted models may therefore be conservative. Finally, other weaknesses include limited sample size to examine multiple joint strata of potential modifiers (eg, sex and age), the assumption of equal weighting across components in our financial well-being index (which may not reflect their true relative importance), and insufficient follow-up time for rigorous temporal evaluation of mediating mechanisms.

Key strengths of our study include creating and validating a multidimensional index of financial well-being that can be examined over time in the HRS, during years when more recently developed scales like the CFPB-FWB Scale are unavailable (eg, 2004-2018). Results of multiple validation analyses suggested that our index adequately captures the construct of financial well-being. Our longitudinal study design also enabled evaluation of average financial well-being and changes in financial well-being over time and their subsequent association with memory function and decline, and we had sufficient sample size to compare associations in younger vs older adults. Finally, we used a previously validated composite memory outcome that accounted for memory outcomes reported by proxy, which helps reduce potential attrition based on the outcome (by retaining participants in the sample who had severe enough cognitive impairment that it prevented their completion of memory assessments).

Altogether, our findings support the hypothesis that financial hardship, particularly worsening financial well-being in midlife and older age, negatively impacts brain health. Future studies should investigate the potential later-life cognitive benefits of interventions that improve older adults’ financial circumstances or buffer the impact of later-life financial changes.^49^ This includes exploring earlier-life interventions that enhance financial security before retirement, as well as those providing supplemental income support during later-life financial shifts.

## Supplementary Material

Web_Material_kwag054

## Data Availability

All data are publicly available, and we cite all relevant data sources and coding programs/packages in the manuscript. We provide tables describing relevant variable names, their location in the datasets, and how we operationalized them in analyses, enabling replication of our study results. Analytic code can be found at https://github.com/klkezios/fwb-and-memory
