# Supplementary material for: Changes in financial well-being and memory function and decline in middle-aged and older adults
Source: Am J Epidemiol. 2026 Mar 16;195(7):1850–8. doi: 10.1093/aje/kwag054 (PMC13343384; doi:10.1093/aje/kwag054)
Supplement: Web_Material_kwag054 [file web_material_kwag054.docx]

**Supplementary Data File** for **“**Changes in financial well-being and memory function and decline in middle-aged and older adults”

**Authors:** Katrina L. Kezios, Jordan Vo, Zihan Chen, Sarah Weber, Allison E. Aiello, Adina Zeki Al Hazzouri

**List of Included Materials:**

**Appendix S1**. Supplemental methods for validating our financial well-being index

- Table S1a. Original question wording and answer choices of FWB items in 2020 HRS survey
- Table S1b. Availability of financial well-being index items and scales across survey waves in HRS

**Appendix S2**: Supplementary descriptive analyses

- Figure S1. Sample selection flowchart
- Table S2a. Baseline characteristics of the analytic sample of Health and Retirement Study participants, stratified by exposure (improvement of financial well-being vs. not) and age (51-64 vs. ≥65 years)
- Table S2b. Baseline characteristics of the analytic sample of Health and Retirement Study participants, stratified by exposure (worsening of financial well-being vs. not) and age (51-64 vs. ≥65 years)
- Table S2c. Distribution of sample baseline characteristics with progressive inclusion/restriction criteria applied (overall sample)
- Table S2d. Distribution of sample baseline characteristics with progressive inclusion/restriction criteria applied (participants aged 51-64 years)
- Table S2e. Distribution of sample baseline characteristics with progressive inclusion/restriction criteria applied (participants aged ≥65 years)

**Appendix S3**: Results of validation analyses for financial well-being index

- Table S3a. Summary statistics for FWB index scores and CFPB-FWB scale scores
- Table S3b. Median (25th%, 75th%) CFPB-FWB scores within levels of each binary FWB index item
- Table S3c. Bivariate associations between FWB index scores and both FWB index and CFPB-FWB z-scores and four validation outcomes in the Health and Retirement Study sample
- Figure S2. Spearman correlations between FWB index items and summary scores (rows) and CFPB-FWB scale items and summary scores (columns)

**Appendix S4**: Regression and sensitivity analysis results

- Table S4a. Confounder-adjusted associations between financial well-being exposures and memory function z-scores in 2016 and memory decline from 2016-2020 from sequentially adjusted models.
- Table S4b. Sensitivity analysis addressing reverse causation by removing all participants with poor memory function at study baseline (2010/2012).
- Table S4c. Sensitivity analysis using changes in memory function from study baseline as outcome in 2016-2020.
- Table S4d. Sensitivity analysis additionally adjusting Model 4 (fully confounder-adjusted) for changes in marital status, employment status, BMI, and health status from exposure baseline (2010/2012) to exposure follow-up (2014/2016).
- Table S4e. Sensitivity analysis comparing unweighted Model 4 (original) with weighted Model 4 that accounts for attrition and missing data

**Appendix S1**. Supplemental methods for validating our financial well-being index

We validated our constructed financial well-being index by comparing 2020 HRS participants’ scores on our index with their scores on the Consumer Financial Protection Bureau’s Financial Well-Being (CFPB-FWB) scale. For validation analyses, we focused on a subsample of 4,688 participants who participated in the 2020 survey wave and were invited to and completed the psychosocial leave behind questionnaire [1-3]. To align with CFPB scoring procedures, we required participants have complete data on all items in the CFPB-FWB scale and our financial well-being index. We additionally excluded anyone whose leave behind questionnaire was reported as completed by someone other than the participant and anyone aged ≤50, for a sample size of 4,011 for all comparisons of the two financial well-being measures.

Poor financial well-being index

As summarized in our manuscript, we developed an index of poor financial well-being (“FWB index”) from indicators of financial hardship addressing similar dimensions to CFPB-FWB items. We prioritized indicators collected at multiple HRS surveys over time. Indicators were based on participants’ subjective assessments of their finances (*financial dissatisfaction, low financial control, difficulty paying bills, ongoing financial strain,* and *taking less medication because of money)* or objective financial indicators (*having low income, having no housing wealth*, and *having low non-housing wealth*). The original items and their answer choices are in **Table S1**. Except in one instance (financial strain in 2008) all items were collected at each biennial HRS survey from 2004 to present (**Table S2**). For this analysis, items are located in version 2 of the publicly available 2020 HRS RAND longitudinal file [4] and the 2020 HRS CORE dataset sections “N” and “LB” [5].

CFPB-FWB scale (convergent validity measure)

The CFPB-FWB scale comprises two parts and 10 total questions. Participants were first asked if the following statements describe them: 1) *I could handle a major unexpected expense*, 2) *I am securing my financial future*, 3) *Because of my money situation, I feel like I will never have the things I want in life*, 4) *I can enjoy life because of the way I’m managing my money*, 5) *I am just getting by financially*, and 6) *I am concerned that the money I have or will save won’t last.* Participants reported if the statements described them “Completely”, “Very Well”, “Somewhat”, “Very Little”, or “Not at all”. Responses to questions 1, 2, and 4 were assigned values of 4 (“Completely”) through 0 (“Not at all”). Responses to questions 3, 5, and 6 were scored in reverse.

Participants were then asked if the following statements applied to them: 7) *Giving a gift for a wedding, birthday or other occasion would put a strain on my finances for the month*, 8) *I have money left over at the end of the month*, 9) *I am behind with my finances*, and 10) *My finances control my life*. Participants reported if the statements applied to them “Always”, “Often”, “Sometimes”, “Rarely”, or “Never”. Responses to questions 7, 9, and 10 were assigned values of 0 (“Always”) through 4 (“Never”). Responses to question 8 were scored in reverse.

Raw scores were computed by summing all items (sample min-max: 0-40), and final scores were calculated according to CFPB-FWB's table-based scoring instructions [6, 7], which scales raw scores based on age (<62 vs. ≥62) and mode of scale administration. We assumed the scale was self-administered for all participants whose leave behind questionnaire was not reported as being completed by someone other than the participant. Final scores in our sample ranged from 14 to 95; higher scores indicate *better* financial well-being.

Financial literacy (discriminant validity measure)

In a small subsample of HRS participants who met our sample criteria and additionally completed an experimental module on retirement knowledge in 2020 (module 1, N=340), we evaluated discriminant validity by comparing correlations between FWB index scores and scores for “financial literacy” [8, 9].

Financial literacy (possessing knowledge and understanding of and demonstrating skills in financial matters) was measured using the Big Three financial literacy questions developed by Lusardi and Mitchell (2011) [8]. These questions were designed to assess one’s knowledge of interest compounding, inflation, and risk diversification [9]. The three questions and their correct answer choices are as follows:

- The question assessing understanding of interest compounding asked: *“Suppose you had $100 in a savings account and the interest rate was 2% per year. After 5 years, how much do you think you would have in the account if you left the money to grow?”* Answer choices were “more than $102” (*correct*), “exactly $102”, “less than $102”, and “do not know”.
- The question assessing understanding of inflation asked: *“Imagine that the interest rate on your savings account was 1% per year and inflation was 2% per year. After 1 year, would you be able to buy?”* Answer choices were “more than today”, “exactly the same as today”, “less than today” (*correct*), and “do not know”.
- The questions assessing understanding of risk diversification asked: “*Do you think that the following statement is true or false? “Buying a single company stock usually provides a safer return than a stock mutual fund*.” Answer choices were “true”, “false” (*correct*), and “do not know”.

For all three questions, correct responses were assigned a value of 1 and all other answer choices (incorrect responses or do not know) were assigned 0. We defined financial literacy as correctly answering all three questions; 37% of participants in this subsample were financially literate by this definition. Further, in this subsample of 340 participants, median [IQR] scores for our FWB index (0 [2]) and the CFPB-FWB scale (61 [19]) were similar to median [IQR] scores in the full study sample (1 [2] and 62 [17], respectively).

Validation outcomes

We examined associations between both financial well-being measures and four validation outcomes: i) fair/poor self-rated health, ii) receiving Medicaid, iii) reporting income from government assistance, and iv) food insecurity. *Self-rated health*: Participants were asked “Would you say your health is excellent, very good, good, fair, or poor?”. Responses were categorized as fair/poor vs. good/very good/excellent. *Receiving Medicaid*: participants were asked if they were covered by Medicaid at any time since their last interview (yes vs. no). *Reporting income from government assistance*: Participants reported the value of income received from veteran’s benefits, welfare, and/or food stamps (herein, “government assistance”) in the last calendar year (a non-zero value was coded as yes vs. no). *Food insecurity*: A subset of participants (financial respondents) [5] reported if since their last interview they “always had enough money to buy the food they need”; those who did not respond “Yes” were further asked if in the past 12 months they “ever ate less than they felt they should because there wasn’t enough money to buy food”. Participants who answered “no” to the first and “yes” to the second question were defined as food insecure vs. food secure. Participants missing outcome data were excluded from analyses, leaving N=4,009, N=3,990, and N=2,855 participants for self-rated health, Medicaid receipt status, or food insecurity analyses, respectively.

Statistical Analyses

*Scale-level analyses (convergent and discriminant validity)*

To assess how well participants’ FWB index scores aligned with their scores on the CFPB’s validated FWB scale, we reported distributions of CFPB-FWB scores within levels of FWB index scores, and we examined the Spearman correlation between the two measures. We considered evidence of convergent validity as a correlation >|0.5| [10]. We also created quintiles of CFPB-FWB scores and reported the percentage of participants with a given FWB index score in a given CFPB-FWB quintile.

We assessed discriminant validity by examining Spearman correlations between participant’s FWB index scores and financial literacy [8]—a related but distinct construct from financial well-being [9, 11]—among a subsample of participants with data on both (N=340). Specifically, we examined Spearman correlations between summary scores on our FWB index and correctly responding to all three financial literacy questions as well as providing correct responses for the individual questions. We considered correlations for discriminant measures (financial literacy) that were noticeably lower than correlations for the convergent measure (CFPB-FWB) as providing evidence of discriminant validity [12]. For comparison, we also evaluated these correlations using CFPB-FWB scores to ensure discriminant validity was also observed for the gold standard measure.

*Item-level analyses (content validity and internal consistency)*

To evaluate if our FWB index adequately captured content represented in the CFPB-FWB scale, we examined intra-item correlations between items comprising both measures [13]. We also examined Spearman correlations between items in our index and CFPB-FWB summary scores, with correlations >|0.30| considered good [14]. For descriptive purposes, we additionally reported the median and interquartile range of CFPB-FWB summary scores for each FWB index item. Finally, we evaluated the internal consistency of items in our FWB index using Cronbach’s alpha, considering a value of 0.70-0.90 to indicate good internal consistency [15-17].

*Validation outcomes (construct validity)*

Finally, we examined associations between FWB index scores and the four validation outcomes using logistic regression models [13, 18]. We then assessed if the magnitudes of associations produced by our FWB index were similar to those produced by the CFPB-FWB scale by creating sample-specific z-scored versions of each FWB measure and comparing the magnitudes of their associations for each validation outcome. For these comparisons, CFPB-FWB scores were reverse coded (by subtracting scores from 96 prior to z-scoring) so that, for both measures, higher scores meant worse financial well-being. All analyses were conducted in R version 4.1.0 (R Core Team, 2021) [19-21].

**Table S1a**. Original question wording and answer choices of FWB items in 2020 HRS survey

| **Financial indicators in FWB index** | **2020 HRS Survey question wording** |
| --- | --- |
| Low control over finances | Using a 0 to 10 scale where 0 means “no control at all” and 10 means “very much control”, how would you rate the amount of control you have over your financial situation these days? |
| Low financial satisfaction | Please think about your life and situation RIGHT NOW. How satisfied are you with your present financial situation?   1. Completely satisfied 2. Very satisfied 3. Somewhat satisfied 4. Not very satisfied 5. Not at all satisfied |
| Difficulty paying bills | How difficult is it for (you/your family) to meet monthly payments on (your/your family’s) bills?   1. Not at all difficult 2. Not very difficult 3. Somewhat difficult 4. Very difficult 5. Completely difficult |
| Ongoing financial strain that results in distress | Please read the list below and indicate whether or not any of these are current and ongoing problems that have lasted twelve months or longer. If the problem is happening to you, indicate how upsetting it has been. Check the answer that is most like your current situation.  Ongoing financial strain   1. No, it didn’t happen 2. Yes, but not upsetting 3. Yes, somewhat upsetting 4. Yes, very upsetting |
| Taking less medication because of cost | Sometimes people delay taking medication or filling prescriptions because of the cost. At any time [since the last interview month/year or in the last two years] have you ended up taking less medication than was prescribed for you because of the cost?  1. Yes  5. No |
| Low income | Family income <125% the federal poverty line for a family of four. Household income variable is H15ITOT from RAND, equivalized for household size and then converted to income for a family of four |
| No housing wealth | A value of ≤0 for housing wealth (variable H15ATOTH in RAND longitudinal file: “net value of primary residence”, which incorporates 0 values for non-homeowners). |
| Low non-housing wealth | Wealth in the bottom quartile of the sample (using variable H15ATOTN in RAND longitudinal file: “total non-housing wealth”). |

**Table S1b**. Availability of financial well-being index items and scales across survey waves in HRS

|  | **Available survey year^1^** | | | | | | | | | |
| --- | --- | --- | --- | --- | --- | --- | --- | --- | --- | --- |
| **Financial well-being indicator/scale** | **2004** | **2006** | **2008** | **2010** | **2012** | **2014** | **2016** | **2018** | **2020** | **2022** |
| Financial satisfaction | LB | LB | LB | LB | LB | LB | LB | LB | LB | LB |
| Difficulty paying bills | LB | LB | LB | LB | LB | LB | LB | LB | LB | LB |
| Ongoing financial strain | LB | LB | *n/a* | LB | LB | LB | LB | LB | LB | LB |
| Taking less medication because of cost | N | N | N | N | N | N | N | N | N | N |
| Income | RAND | RAND | RAND | RAND | RAND | RAND | RAND | RAND | RAND | *** |
| Total housing wealth | RAND | RAND | RAND | RAND | RAND | RAND | RAND | RAND | RAND | *** |
| Total non-housing wealth | RAND | RAND | RAND | RAND | RAND | RAND | RAND | RAND | RAND | *** |
| CFPB Financial well-being scale | *n/a* | *n/a* | *n/a* | *n/a* | *n/a* | *n/a* | *n/a* | *n/a* | LB | LB |

^1^We begin reporting in 2004 because this was the first year the leave behind questionnaire was included in the survey. Different HRS subsamples contribute to different years (a subset of participants have some longitudinal data on these measures).

n/a = not collected in a given year; * = not yet released; LB = item available in psychosocial leave behind questionnaire; N = item available in section N of CORE data files; RAND = item available in longitudinal RAND file.

­­­­

**Appendix S2**: Supplementary descriptive analyses

**Figure S1**. Sample selection flowchart.


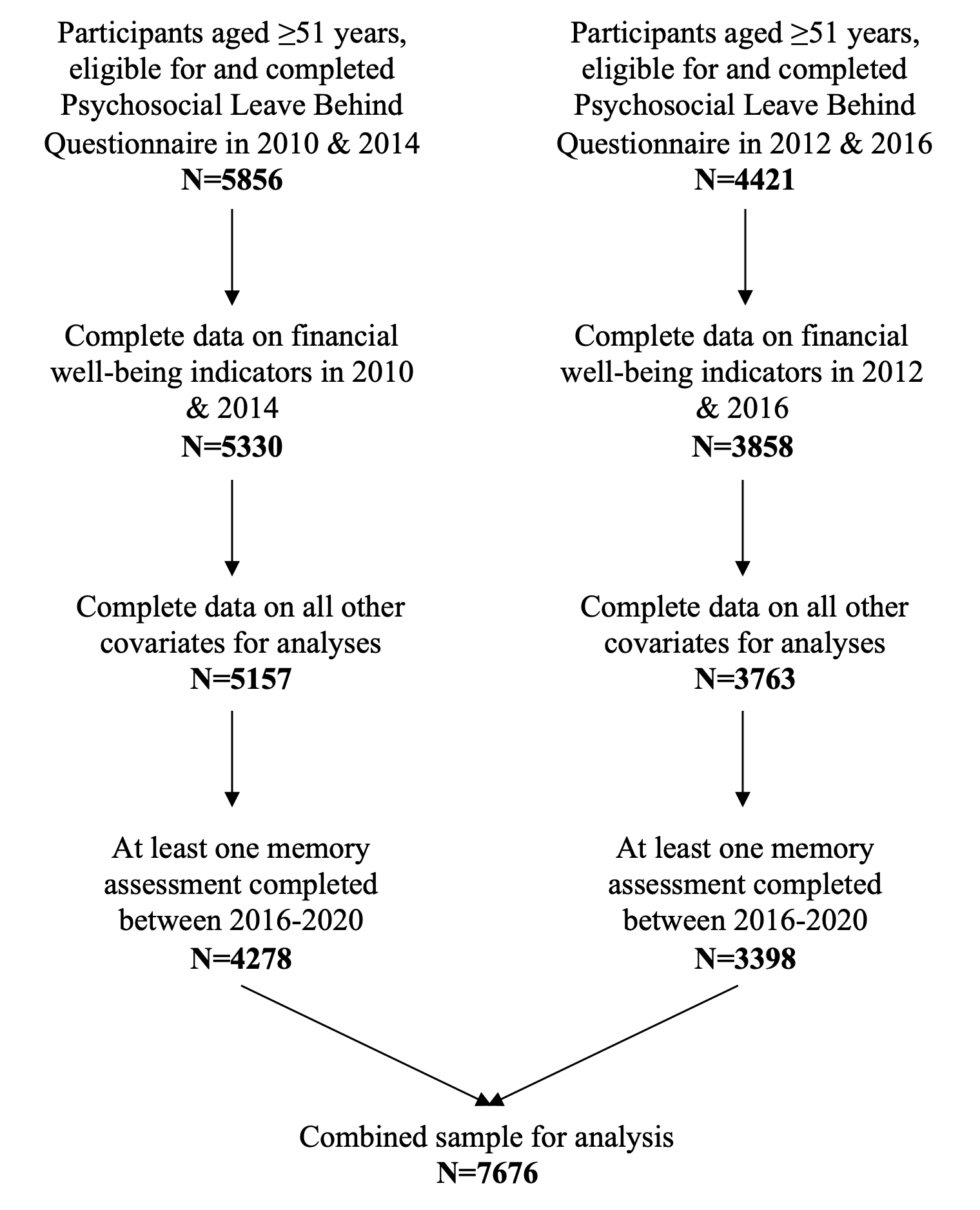


**Figure S1 Legend.** Sample selection flowchart based on data availability i) at exposure baseline (2010 or 2012, depending on HRS subsample), ii) at exposure follow-up (2014 or 2016, depending on HRS subsample), and iii) over outcome follow-up (2016-2020 for both subsamples).

**Table S2a**. Baseline characteristics of the analytic sample of Health and Retirement Study participants, stratified by exposure (improvement of financial well-being vs. not) and age (51-64 vs. ≥65 years)

|  | **Age group** | | | | |
| --- | --- | --- | --- | --- | --- |
|  | **51-64** | |  | **≥65** | |
| **Baseline characteristic^*^** | **FWB**  **improved** | **FWB**  **did not improve** |  | **FWB**  **improved** | **FWB**  **did not improve** |
|  | **(N=601)** | **(N=3007)** |  | **(N=278)** | **(N=3790)** |
| **Age at baseline (years)** | |  |  |  |  |
| Mean (SD) | 57.1 (3.74) | 57.8 (3.83) |  | 72.4 (5.83) | 73.9 (5.94) |
| Median [Q1-Q3] | 57.0 [54.0-60.0] | 58.0 [55.0-61.0] |  | 71.5 [68.0-76.0] | 73.0 [69.0-78.0] |
| **Race¹** |  |  |  |  |  |
| Black or other | 205 (34.1%) | 766 (25.5%) |  | 57 (20.5%) | 471 (12.4%) |
| White | 396 (65.9%) | 2241 (74.5%) |  | 221 (79.5%) | 3319 (87.6%) |
| **Gender²** |  |  |  |  |  |
| Female | 369 (61.4%) | 1814 (60.3%) |  | 185 (66.5%) | 2224 (58.7%) |
| Male | 232 (38.6%) | 1193 (39.7%) |  | 93 (33.5%) | 1566 (41.3%) |
| **Years of education** | |  |  |  |  |
| Mean (SD) | 13.4 (2.29) | 13.9 (2.34) |  | 12.8 (2.45) | 13.2 (2.52) |
| Median [Q1-Q3] | 13.0 [12.0-15.0] | 14.0 [12.0-16.0] |  | 12.0 [12.0-14.0] | 12.0 [12.0-16.0] |
| **Born in a Southern US state** | |  |  |  |  |
| Yes | 179 (29.8%) | 863 (28.7%) |  | 104 (37.4%) | 1181 (31.2%) |
| No | 422 (70.2%) | 2144 (71.3%) |  | 174 (62.6%) | 2609 (68.8%) |
| **Parental years of education³** | |  |  |  |  |
| <8 years | 45 (7.5%) | 196 (6.5%) |  | 46 (16.5%) | 465 (12.3%) |
| 8-12 years | 357 (59.4%) | 1749 (58.2%) |  | 179 (64.4%) | 2385 (62.9%) |
| >12 years | 157 (26.1%) | 940 (31.3%) |  | 38 (13.7%) | 779 (20.6%) |
| Unknown | 42 (7.0%) | 122 (4.1%) |  | 15 (5.4%) | 161 (4.2%) |
| **Marital status** |  |  |  |  |  |
| Married/partnered | 375 (62.4%) | 2173 (72.3%) |  | 159 (57.2%) | 2527 (66.7%) |
| Separated/divorced/widowed | 163 (27.1%) | 646 (21.5%) |  | 115 (41.4%) | 1174 (31.0%) |
| Never married | 63 (10.5%) | 188 (6.3%) |  | 4 (1.4%) | 89 (2.3%) |
| **Has employer-provided health insurance** | | |  |  |  |
| Yes | 330 (54.9%) | 2105 (70.0%) |  | 68 (24.5%) | 1295 (34.2%) |
| No | 271 (45.1%) | 902 (30.0%) |  | 210 (75.5%) | 2495 (65.8%) |
| **Occupational skill level** | |  |  |  |  |
| Higher skill | 111 (18.5%) | 857 (28.5%) |  | 16 (5.8%) | 285 (7.5%) |
| Lower skill | 183 (30.4%) | 942 (31.3%) |  | 42 (15.1%) | 469 (12.4%) |
| Not working | 266 (44.3%) | 1039 (34.6%) |  | 217 (78.1%) | 3008 (79.4%) |
| Unknown | 41 (6.8%) | 169 (5.6%) |  | 3 (1.1%) | 28 (0.7%) |
| **Wealth at baseline (in 2010 dollars)⁴** | | |  |  |  |
| Mean (SD) | 143000 (461000) | 458000 (910000) |  | 220000 (457000) | 577000 (848000) |
| Median [Q1-Q3] | 25200 [0-153000] | 184000 [40100-518000] |  | 90800 [5840-243000] | 303000 [110000-714000] |
| **Income at baseline (in 2010 dollars)⁵** | | |  |  |  |
| Mean (SD) | 54300 (56100) | 94800 (109000) |  | 41100 (53700) | 62500 (82900) |
| Median [Q1-Q3] | 39800 [16300-72300] | 67900 [34000-118000] |  | 27800 [16100-47700] | 41800 [25400-72200] |
| **Alcohol drinking status** | |  |  |  |  |
| Ever drinks alcohol | 369 (61.4%) | 2005 (66.7%) |  | 129 (46.4%) | 2108 (55.6%) |
| Never drinks alcohol | 232 (38.6%) | 1002 (33.3%) |  | 149 (53.6%) | 1682 (44.4%) |
| **Smoking status** | |  |  |  |  |
| Currently smokes cigarettes | 147 (24.5%) | 509 (16.9%) |  | 32 (11.5%) | 248 (6.5%) |
| Never/formerly smoked cigarettes | 454 (75.5%) | 2498 (83.1%) |  | 246 (88.5%) | 3542 (93.5%) |
| **Body Mass Index** | |  |  |  |  |
| Mean (SD) | 30.3 (6.60) | 29.3 (6.42) |  | 28.7 (6.56) | 28.3 (5.57) |
| Median [Q1-Q3] | 29.0 [25.7-34.2] | 28.2 [24.8-32.6] |  | 27.7 [24.1-31.8] | 27.5 [24.4-31.3] |
| **No. of doctor diagnosed health conditions** | | |  |  |  |
| Mean (SD) | 1.79 (1.40) | 1.52 (1.33) |  | 2.61 (1.49) | 2.25 (1.30) |
| Median [Q1-Q3] | 2.00 [1.00-3.00] | 1.00 [0-2.00] |  | 2.00 [2.00-4.00] | 2.00 [1.00-3.00] |
| **Depressive symptoms (CESD-8 score ≥4)** | | |  |  |  |
| Yes | 131 (21.8%) | 365 (12.1%) |  | 65 (23.4%) | 279 (7.4%) |
| No | 470 (78.2%) | 2642 (87.9%) |  | 213 (76.6%) | 3511 (92.6%) |
| **Average FWB score over time** | | |  |  |  |
| Mean (SD) | 2.83 (1.45) | 1.46 (1.80) |  | 2.49 (1.34) | 0.904 (1.32) |
| Median [Q1-Q3] | 2.50 [1.50-4.00] | 0.500 [0-2.50] |  | 2.00 [1.00-3.50] | 0.500 [0-1.50] |
| **Marriage/partnership ended** | |  |  |  |  |
| Yes | 35 (5.8%) | 137 (4.6%) |  | 24 (8.6%) | 283 (7.5%) |
| No | 566 (94.2%) | 2870 (95.4%) |  | 254 (91.4%) | 3507 (92.5%) |
| **Became unemployed** | |  |  |  |  |
| Yes | 18 (3.0%) | 92 (3.1%) |  | 5 (1.8%) | 58 (1.5%) |
| No | 583 (97.0%) | 2915 (96.9%) |  | 273 (98.2%) | 3732 (98.5%) |
| **Experienced significant weight loss** | | |  |  |  |
| Yes | 69 (11.5%) | 263 (8.7%) |  | 37 (13.3%) | 403 (10.6%) |
| No | 527 (87.7%) | 2729 (90.8%) |  | 237 (85.3%) | 3371 (88.9%) |
| Missing | 5 (0.8%) | 15 (0.5%) |  | 4 (1.4%) | 16 (0.4%) |
| **Experienced ≥1 newly diagnosed health condition** | | | | |  |
| Yes | 178 (29.6%) | 868 (28.9%) |  | 80 (28.8%) | 1139 (30.1%) |
| No | 423 (70.4%) | 2139 (71.1%) |  | 198 (71.2%) | 2651 (69.9%) |
| **Composite memory z-score at baseline** | | |  |  |  |
| Mean (SD) | 0.689 (0.468) | 0.721 (0.446) |  | 0.0564 (0.725) | 0.0355 (0.786) |
| Median [Q1-Q3] | 0.763 [0.403-1.01] | 0.774 [0.488-1.00] |  | 0.123 [-0.314-0.583] | 0.170 [-0.367-0.587] |
| Missing | 0 (0%) | 4 (0.1%) |  | 1 (0.4%) | 17 (0.4%) |
| No. = Number; CESD-8 = 8-item version of the Center for Epidemiologic Studies Depression Scale; FWB=financial well-being  ^*^Baseline is the year of first financial well-being measure, either 2010 or 2012 in the combined sample  ¹Self-reported by participants and collapsed into categories of Black, White, or Other racial group  ²Self-reported by participants as male or female (no distinction in surveys between sex/gender)  ³Highest of either parents reported years of education  ⁴Sum of all sources of household assets minus debts reported in 2010 dollars  ⁵Sum of all sources of household income reported in 2010 dollars | | | | | |

**Table S2b**. Baseline characteristics of the analytic sample of Health and Retirement Study participants, stratified by exposure (worsening of financial well-being vs. not) and age (51-64 vs. ≥65 years)

|  | **Age group** | | | | |
| --- | --- | --- | --- | --- | --- |
|  | **51-64** | |  | **≥65** | |
| **Baseline characteristic^*^** | **FWB**  **worsened** | **FWB**  **did not worsen** |  | **FWB**  **worsened** | **FWB**  **did not worsen** |
|  | **(N=275)** | **(N=3333)** |  | **(N=284)** | **(N=3784)** |
| **Age at baseline (years)** | |  |  |  |  |
| Mean (SD) | 57.3 (3.77) | 57.7 (3.83) |  | 73.7 (6.12) | 73.8 (5.93) |
| Median [Q1-Q3] | 57.0 [54.0-61.0] | 58.0 [54.0-61.0] |  | 73.0 [69.0-77.0] | 73.0 [69.0-78.0] |
| **Race¹** |  |  |  |  |  |
| Black or other | 86 (31.3%) | 885 (26.6%) |  | 48 (16.9%) | 480 (12.7%) |
| White | 189 (68.7%) | 2448 (73.4%) |  | 236 (83.1%) | 3304 (87.3%) |
| **Gender²** |  |  |  |  |  |
| Female | 181 (65.8%) | 2002 (60.1%) |  | 195 (68.7%) | 2214 (58.5%) |
| Male | 94 (34.2%) | 1331 (39.9%) |  | 89 (31.3%) | 1570 (41.5%) |
| **Years of education** | |  |  |  |  |
| Mean (SD) | 13.2 (2.46) | 13.8 (2.32) |  | 12.5 (2.64) | 13.3 (2.50) |
| Median [Q1-Q3] | 13.0 [12.0-15.0] | 14.0 [12.0-16.0] |  | 12.0 [12.0-14.0] | 12.0 [12.0-16.0] |
| **Born in a Southern US state** | |  |  |  |  |
| Yes | 102 (37.1%) | 940 (28.2%) |  | 105 (37.0%) | 1180 (31.2%) |
| No | 173 (62.9%) | 2393 (71.8%) |  | 179 (63.0%) | 2604 (68.8%) |
| **Parental years of education³** | |  |  |  |  |
| <8 years | 29 (10.5%) | 212 (6.4%) |  | 51 (18.0%) | 460 (12.2%) |
| 8-12 years | 165 (60.0%) | 1941 (58.2%) |  | 176 (62.0%) | 2388 (63.1%) |
| >12 years | 65 (23.6%) | 1032 (31.0%) |  | 43 (15.1%) | 774 (20.5%) |
| Unknown | 16 (5.8%) | 148 (4.4%) |  | 14 (4.9%) | 162 (4.3%) |
| **Marital status** |  |  |  |  |  |
| Married/partnered | 180 (65.5%) | 2368 (71.0%) |  | 174 (61.3%) | 2512 (66.4%) |
| Separated/divorced/widowed | 75 (27.3%) | 734 (22.0%) |  | 102 (35.9%) | 1187 (31.4%) |
| Never married | 20 (7.3%) | 231 (6.9%) |  | 8 (2.8%) | 85 (2.2%) |
| **Has employer-provided health insurance** | | |  |  |  |
| Yes | 175 (63.6%) | 2260 (67.8%) |  | 74 (26.1%) | 1289 (34.1%) |
| No | 100 (36.4%) | 1073 (32.2%) |  | 210 (73.9%) | 2495 (65.9%) |
| **Occupational skill level** | |  |  |  |  |
| Higher skill | 61 (22.2%) | 907 (27.2%) |  | 12 (4.2%) | 289 (7.6%) |
| Lower skill | 93 (33.8%) | 1032 (31.0%) |  | 43 (15.1%) | 468 (12.4%) |
| Not working | 101 (36.7%) | 1204 (36.1%) |  | 223 (78.5%) | 3002 (79.3%) |
| Missing | 20 (7.3%) | 190 (5.7%) |  | 6 (2.1%) | 25 (0.7%) |
| **Wealth at baseline (in 2010 dollars)⁴** | | |  |  |  |
| Mean (SD) | 149000 (256000) | 427000 (888000) |  | 222000 (359000) | 577000 (852000) |
| Median  [Q1-Q3] | 69400  [14600-194000] | 159000  [20900-481000] |  | 115000  [30600-238000] | 303000  [107000-715000] |
| **Income at baseline (in 2010 dollars)⁵** | | |  |  |  |
| Mean (SD) | 71800 (109000) | 89400 (102000) |  | 42200 (45800) | 62500 (83300) |
| Median  [Q1-Q3] | 52700  [27500-81000] | 63300  [29900-113000] |  | 31000  [20200-47800] | 41700  [25000-72300] |
| **Alcohol drinking status** | |  |  |  |  |
| Ever drinks alcohol | 164 (59.6%) | 2210 (66.3%) |  | 122 (43.0%) | 2115 (55.9%) |
| Never drinks alcohol | 111 (40.4%) | 1123 (33.7%) |  | 162 (57.0%) | 1669 (44.1%) |
| **Smoking status** | |  |  |  |  |
| Currently smokes cigarettes | 73 (26.5%) | 583 (17.5%) |  | 20 (7.0%) | 260 (6.9%) |
| Never/formerly smoked cigarettes | 202 (73.5%) | 2750 (82.5%) |  | 264 (93.0%) | 3524 (93.1%) |
| **Body Mass Index** | |  |  |  |  |
| Mean (SD) | 31.1 (7.21) | 29.3 (6.38) |  | 29.8 (6.70) | 28.3 (5.54) |
| Median [Q1-Q3] | 30.2 [25.8-34.8] | 28.2 [24.9-32.7] |  | 28.7 [25.5-33.4] | 27.5 [24.4-31.2] |
| **No. of doctor diagnosed health conditions** | | |  |  |  |
| Mean (SD) | 1.86 (1.44) | 1.54 (1.34) |  | 2.73 (1.35) | 2.24 (1.31) |
| Median [Q1-Q3] | 2.00 [1.00-3.00] | 1.00 [1.00-2.00] |  | 3.00 [2.00-4.00] | 2.00 [1.00-3.00] |
| **Depressive symptoms (CESD-8 score ≥4)** | | |  |  |  |
| Yes | 50 (18.2%) | 446 (13.4%) |  | 35 (12.3%) | 309 (8.2%) |
| No | 225 (81.8%) | 2887 (86.6%) |  | 249 (87.7%) | 3475 (91.8%) |
| **Average FWB score over time** | | |  |  |  |
| Mean (SD) | 2.76 (1.43) | 1.60 (1.82) |  | 2.44 (1.40) | 0.905 (1.32) |
| Median [Q1-Q3] | 2.50 [1.50-3.50] | 1.00 [0-2.50] |  | 2.00 [1.00-3.50] | 0.500 [0-1.50] |
| **Marriage/partnership ended** | |  |  |  |  |
| Yes | 34 (12.4%) | 138 (4.1%) |  | 36 (12.7%) | 271 (7.2%) |
| No | 241 (87.6%) | 3195 (95.9%) |  | 248 (87.3%) | 3513 (92.8%) |
| **Became unemployed** | |  |  |  |  |
| Yes | 17 (6.2%) | 93 (2.8%) |  | 6 (2.1%) | 57 (1.5%) |
| No | 258 (93.8%) | 3240 (97.2%) |  | 278 (97.9%) | 3727 (98.5%) |
| **Experienced significant weight loss** | | |  |  |  |
| Yes | 41 (14.9%) | 291 (8.7%) |  | 34 (12.0%) | 406 (10.7%) |
| No | 231 (84.0%) | 3025 (90.8%) |  | 247 (87.0%) | 3361 (88.8%) |
| Missing | 3 (1.1%) | 17 (0.5%) |  | 3 (1.1%) | 17 (0.4%) |
| **Experienced ≥1 newly diagnosed health condition** | | | | |  |
| Yes | 98 (35.6%) | 948 (28.4%) |  | 91 (32.0%) | 1128 (29.8%) |
| No | 177 (64.4%) | 2385 (71.6%) |  | 193 (68.0%) | 2656 (70.2%) |
| **Composite memory z-score at baseline** | | |  |  |  |
| Mean (SD) | 0.665 (0.463) | 0.720 (0.448) |  | -0.0825 (0.865) | 0.0459 (0.775) |
| Median [Q1-Q3] | 0.750 [0.356-0.962] | 0.774 [0.483-1.01] |  | 0.0548 [-0.548-0.586] | 0.170 [-0.348-0.586] |
| Missing | 1 (0.4%) | 3 (0.1%) |  | 1 (0.4%) | 17 (0.4%) |
| No. = Number; CESD-8 = 8-item version of the Center for Epidemiologic Studies Depression Scale; FWB=financial well-being  ^*^Baseline is the year of first financial well-being measure, either 2010 or 2012 in the combined sample  ¹Self-reported by participants and collapsed into categories of Black, White, or Other racial group  ²Self-reported by participants as male or female (no distinction in surveys between sex/gender)  ³Highest of either parents reported years of education  ⁴Sum of all sources of household assets minus debts reported in 2010 dollars  ⁵Sum of all sources of household income reported in 2010 dollars | | | | | |

**Table S2c**. Distribution of sample baseline characteristics with progressive inclusion/restriction criteria applied (overall sample)

|  | **Overall Sample** | | | |
| --- | --- | --- | --- | --- |
|  | **Completed SAQ**  **and age ≥51**  **N=10277** | **Complete data**  **on FWB items**  **N=9188** | **Complete data**  **on covariates**  **N=8920** | **At least one memory outcome 2016-2020**  **N=7676** |
| **Age at baseline (years)** |  |  |  |  |
| Mean (SD) | 66.5 (9.71) | 66.2 (9.61) | 66.2 (9.60) | 66.2 (9.50) |
| Median [Q1-Q3] | 66.0 [58.0-74.0] | 65.0 [58.0-73.0] | 65.0 [58.0-73.0] | 66.0 [58.0-73.0] |
| **Race¹** |  |  |  |  |
| Black or other | 2234 (21.7%) | 1900 (20.7%) | 1847 (20.7%) | 1499 (19.5%) |
| White | 8026 (78.1%) | 7272 (79.1%) | 7073 (79.3%) | 6177 (80.5%) |
| *Missing* | *17 (0.2%)* | *16 (0.2%)* | *-* | *-* |
| **Gender²** |  |  |  |  |
| Female | 6164 (60.0%) | 5461 (59.4%) | 5298 (59.4%) | 4592 (59.8%) |
| Male | 4113 (40.0%) | 3727 (40.6%) | 3622 (40.6%) | 3084 (40.2%) |
| **Years of education** |  |  |  |  |
| Mean (SD) | 13.0 (2.88) | 13.2 (2.79) | 13.2 (2.74) | 13.5 (2.45) |
| Median [Q1-Q3] | 13.0 [12.0-16.0] | 13.0 [12.0-16.0] | 13.0 [12.0-16.0] | 13.0 [12.0-16.0] |
| *Missing* | *2 (0.0%)* | *2 (0.0%)* | *-* | *-* |
| **Born in a Southern US state** |  |  |  |  |
| Yes | 3089 (30.1%) | 2686 (29.2%) | 2603 (29.2%) | 2327 (30.3%) |
| No | 7183 (69.9%) | 6500 (70.7%) | 6317 (70.8%) | 5349 (69.7%) |
| *Missing* | *5 (0.0%)* | *2 (0.0%)* | *-* | *-* |
| **Parental years of education³** |  |  |  |  |
| <8 years | 1508 (14.7%) | 1272 (13.8%) | 1207 (13.5%) | 752 (9.8%) |
| 8-12 years | 5884 (57.3%) | 5328 (58.0%) | 5212 (58.4%) | 4670 (60.8%) |
| >12 years | 2301 (22.4%) | 2110 (23.0%) | 2069 (23.2%) | 1914 (24.9%) |
| Unknown | 584 (5.7%) | 478 (5.2%) | 432 (4.8%) | 340 (4.4%) |
| **Marital status** |  |  |  |  |
| Married/partnered | 6902 (67.2%) | 6291 (68.5%) | 6092 (68.3%) | 5234 (68.2%) |
| Separated/divorced/widowed | 2886 (28.1%) | 2486 (27.1%) | 2432 (27.3%) | 2098 (27.3%) |
| Never married | 487 (4.7%) | 409 (4.5%) | 396 (4.4%) | 344 (4.5%) |
| *Missing* | *2 (0.0%)* | *2 (0.0%)* | *-* | *-* |
| **Has employer-provided health insurance** |  |  |  |  |
| Yes | 4671 (45.5%) | 4336 (47.2%) | 4250 (47.6%) | 3798 (49.5%) |
| No | 5552 (54.0%) | 4810 (52.4%) | 4670 (52.4%) | 3878 (50.5%) |
| *Missing* | *54 (0.5%)* | *42 (0.5%)* | *-* | *-* |
| **Occupational skill level** |  |  |  |  |
| Higher skill | 1489 (14.5%) | 1397 (15.2%) | 1373 (15.4%) | 1269 (16.5%) |
| Lower skill | 2202 (21.4%) | 2016 (21.9%) | 1951 (21.9%) | 1636 (21.3%) |
| Not working | 6264 (61.0%) | 5484 (59.7%) | 5311 (59.5%) | 4530 (59.0%) |
| Unknown | 322 (3.1%) | 291 (3.2%) | 285 (3.2%) | 241 (3.1%) |
| **Wealth at baseline (in 2010 dollars)⁴** |  |  |  |  |
| Mean (SD) | 439000 (803000) | 453000 (820000) | 454000 (818000) | 483000 (848000) |
| Median [Q1-Q3] | 182000 [38700-513000] | 192000 [43200-531000] | 193000 [43700-534000] | 214000 [54300-567000] |
| **Income at baseline (in 2010 dollars)⁵** |  |  |  |  |
| Mean (SD) | 67100 (86700) | 69800 (88900) | 70200 (89500) | 73800 (93100) |
| Median [Q1-Q3] | 43700 [22400-80700] | 46100 [24000-83900] | 46500 [24100-84200] | 49200 [25900-88300] |
| **Drinking status** |  |  |  |  |
| Ever drinks alcohol | 6023 (58.6%) | 5464 (59.5%) | 5324 (59.7%) | 4611 (60.1%) |
| Never drinks alcohol | 4254 (41.4%) | 3724 (40.5%) | 3596 (40.3%) | 3065 (39.9%) |
| **Smoking status** |  |  |  |  |
| Currently smokes cigarettes | 1282 (12.5%) | 1133 (12.3%) | 1111 (12.5%) | 936 (12.2%) |
| Never/formerly smoked cigarettes | 8940 (87.0%) | 8007 (87.1%) | 7809 (87.5%) | 6740 (87.8%) |
| *Missing* | *55 (0.5%)* | *48 (0.5%)* | *-* | *-* |
| **Body Mass Index** |  |  |  |  |
| Mean (SD) | 28.9 (6.04) | 28.9 (6.05) | 28.9 (6.05) | 28.9 (6.07) |
| Median [Q1-Q3] | 27.9 [24.7-32.0] | 27.9 [24.7-32.0] | 28.0 [24.7-32.1] | 27.9 [24.7-32.1] |
| *Missing* | *110 (1.1%)* | *96 (1.0%)* | *-* | *-* |
| **No. of doctor diagnosed health conditions** |  |  |  |  |
| Mean (SD) | 1.98 (1.40) | 1.94 (1.39) | 1.94 (1.39) | 1.94 (1.38) |
| Median [Q1-Q3] | 2.00 [1.00-3.00] | 2.00 [1.00-3.00] | 2.00 [1.00-3.00] | 2.00 [1.00-3.00] |
| **Depressive symptoms (CESD-8 score ≥4)** |  |  |  |  |
| Yes | 1226 (11.9%) | 1062 (11.6%) | 1026 (11.5%) | 840 (10.9%) |
| No | 8979 (87.4%) | 8062 (87.7%) | 7894 (88.5%) | 6836 (89.1%) |
| *Missing* | *72 (0.7%)* | *64 (0.7%)* | *-* | *-* |
| **Composite memory z-score at baseline** |  |  |  |  |
| Mean (SD) | 0.326 (0.766) | 0.326 (0.763) | 0.338 (0.747) | 0.357 (0.730) |
| Median [Q1-Q3] | 0.480 [-0.0306-0.850] | 0.484 [-0.0365-0.841] | 0.488 [-0.0240-0.842] | 0.502 [-0.00469-0.848] |
| *Missing* | *1057 (10.3%)* | *888 (9.7%)* | 810 (9.1%) | *22 (0.3%)* |

**Table S2d**. Distribution of sample baseline characteristics with progressive inclusion/restriction criteria applied (participants aged 51-64 years)

|  | **Participants aged**  **51-64 years** | | | |
| --- | --- | --- | --- | --- |
|  | **Completed SAQ**  **and age ≥51**  **N=10277** | **Complete data**  **on FWB items**  **N=9188** | **Complete data**  **on covariates**  **N=8920** | **At least one memory**  **outcome 2016-2020**  **N=7676** |
| **Age at baseline (years)** |  |  |  |  |
| Mean (SD) | 57.7 (3.80) | 57.6 (3.80) | 57.6 (3.80) | 57.7 (3.82) |
| Median [Q1-Q3] | 58.0 [54.0-61.0] | 58.0 [54.0-61.0] | 58.0 [54.0-61.0] | 58.0 [54.0-61.0] |
| **Race¹** |  |  |  |  |
| Black or other | 1440 (30.2%) | 1256 (28.7%) | 1223 (28.8%) | 971 (26.9%) |
| White | 3314 (69.5%) | 3100 (70.9%) | 3028 (71.2%) | 2637 (73.1%) |
| *Missing* | *17 (0.4%)* | *16 (0.4%)* | *-* | *-* |
| **Gender²** |  |  |  |  |
| Female | 2897 (60.7%) | 2636 (60.3%) | 2557 (60.2%) | 2183 (60.5%) |
| Male | 1874 (39.3%) | 1736 (39.7%) | 1694 (39.8%) | 1425 (39.5%) |
| **Years of education** |  |  |  |  |
| Mean (SD) | 13.3 (2.84) | 13.4 (2.79) | 13.5 (2.71) | 13.8 (2.33) |
| Median [Q1-Q3] | 13.0 [12.0-16.0] | 13.0 [12.0-16.0] | 13.0 [12.0-16.0] | 14.0 [12.0-16.0] |
| *Missing* | 0 (0%) | 0 (0%) |  | *-* |
| **Born in a Southern US state** |  |  |  |  |
| Yes | 1334 (28.0%) | 1190 (27.2%) | 1163 (27.4%) | 1042 (28.9%) |
| No | 3437 (72.0%) | 3182 (72.8%) | 3088 (72.6%) | 2566 (71.1%) |
| *Missing* | 0 (0%) | 0 (0%) |  | *-* |
| **Parental years of education³** |  |  |  |  |
| <8 years | 631 (13.2%) | 542 (12.4%) | 509 (12.0%) | 241 (6.7%) |
| 8-12 years | 2588 (54.2%) | 2402 (54.9%) | 2358 (55.5%) | 2106 (58.4%) |
| >12 years | 1277 (26.8%) | 1197 (27.4%) | 1174 (27.6%) | 1097 (30.4%) |
| Unknown | 275 (5.8%) | 231 (5.3%) | 210 (4.9%) | 164 (4.5%) |
| **Marital status** |  |  |  |  |
| Married/partnered | 3346 (70.1%) | 3115 (71.2%) | 3024 (71.1%) | 2548 (70.6%) |
| Separated/divorced/widowed | 1076 (22.6%) | 957 (21.9%) | 937 (22.0%) | 809 (22.4%) |
| Never married | 348 (7.3%) | 299 (6.8%) | 290 (6.8%) | 251 (7.0%) |
| *Missing* | 1 (0.0%) | 1 (0.0%) |  |  |
| **Has employer-provided health insurance** |  |  |  |  |
| Yes | 2971 (62.3%) | 2796 (64.0%) | 2745 (64.6%) | 2435 (67.5%) |
| No | 1788 (37.5%) | 1568 (35.9%) | 1506 (35.4%) | 1173 (32.5%) |
| *Missing* | 12 (0.3%) | 8 (0.2%) |  |  |
| **Occupational skill level** |  |  |  |  |
| Higher skill | 1128 (23.6%) | 1067 (24.4%) | 1052 (24.7%) | 968 (26.8%) |
| Lower skill | 1542 (32.3%) | 1426 (32.6%) | 1376 (32.4%) | 1125 (31.2%) |
| Not working | 1817 (38.1%) | 1623 (37.1%) | 1571 (37.0%) | 1305 (36.2%) |
| Unknown | 284 (6.0%) | 256 (5.9%) | 252 (5.9%) | 210 (5.8%) |
| **Wealth at baseline (in 2010 dollars)⁴** |  |  |  |  |
| Mean (SD) | 358000 (790000) | 371000 (805000) | 371000 (807000) | 406000 (860000) |
| Median [Q1-Q3] | 113000 [9810-395000] | 122000 [12600-413000] | 123000 [13300-414000] | 146000 [20400-456000] |
| **Income at baseline (in 2010 dollars)⁵** |  |  |  |  |
| Mean (SD) | 79600 (96600) | 82300 (98300) | 83000 (99200) | 88100 (103000) |
| Median [Q1-Q3] | 54500 [23700-102000] | 56700 [25500-104000] | 57100 [25900-105000] | 62500 [29700-111000] |
| **Drinking status** |  |  |  |  |
| Ever drinks alcohol | 3089 (64.7%) | 2851 (65.2%) | 2782 (65.4%) | 2374 (65.8%) |
| Never drinks alcohol | 1682 (35.3%) | 1521 (34.8%) | 1469 (34.6%) | 1234 (34.2%) |
| **Smoking status** |  |  |  |  |
| Currently smokes cigarettes | 870 (18.2%) | 773 (17.7%) | 761 (17.9%) | 656 (18.2%) |
| Never/formerly smoked cigarettes | 3884 (81.4%) | 3582 (81.9%) | 3490 (82.1%) | 2952 (81.8%) |
| *Missing* | 17 (0.4%) | 17 (0.4%) | *-* | *-* |
| **Body Mass Index** |  |  |  |  |
| Mean (SD) | 29.6 (6.46) | 29.5 (6.43) | 29.5 (6.42) | 29.5 (6.46) |
| Median [Q1-Q3] | 28.5 [25.1-32.9] | 28.4 [25.1-32.9] | 28.5 [25.1-32.9] | 28.3 [24.9-32.9] |
| *Missing* | 65 (1.4%) | 56 (1.3%) | *-* |  |
| **No. of doctor diagnosed health conditions** |  |  |  |  |
| Mean (SD) | 1.57 (1.35) | 1.55 (1.34) | 1.55 (1.34) | 1.57 (1.35) |
| Median [Q1-Q3] | 1.00 [1.00-2.00] | 1.00 [1.00-2.00] | 1.00 [1.00-2.00] | 1.00 [1.00-2.00] |
| **Depressive symptoms (CESD-8 score ≥4)** |  |  |  |  |
| Yes | 694 (14.5%) | 623 (14.2%) | 604 (14.2%) | 496 (13.7%) |
| No | 4050 (84.9%) | 3725 (85.2%) | 3647 (85.8%) | 3112 (86.3%) |
| *Missing* | 27 (0.6%) | 24 (0.5%) | *-* | *-* |
| **Composite memory z-score at baseline** |  |  |  |  |
| Mean (SD) | 0.729 (0.444) | 0.715 (0.453) | 0.715 (0.449) | 0.72 (0.45) |
| Median [Q1-Q3] | 0.787 [0.479-1.01] | 0.773 [0.477-1.00] | 0.773 [0.478-1.00] | 0.77 [0.48-1.00] |
| *Missing* | 671 (14.1%) | 580 (13.3%) | 520 (12.2%) | *4 (0.1%)* |

**Table S2e**. Distribution of sample baseline characteristics with progressive inclusion/restriction criteria applied (participants aged ≥65 years)

|  | **Participants aged**  **≥65 years** | | | |
| --- | --- | --- | --- | --- |
|  | **Completed SAQ**  **and age ≥51**  **N=10277** | **Complete data**  **on FWB items**  **N=9188** | **Complete data**  **on covariates**  **N=8920** | **At least one memory**  **outcome 2016-2020**  **N=7676** |
| **Age at baseline (years)** |  |  |  |  |
| Mean (SD) | 74.2 (6.10) | 73.9 (6.06) | 73.9 (6.06) | 73.8 (5.94) |
| Median [Q1-Q3] | 73.0 [69.0-78.0] | 73.0 [69.0-78.0] | 73.0 [69.0-78.0] | 73.0 [69.0-78.0] |
| **Race¹** |  |  |  |  |
| Black or other | 794 (14.4%) | 644 (13.4%) | 624 (13.4%) | 528 (13.0%) |
| White | 4712 (85.6%) | 4172 (86.6%) | 4045 (86.6%) | 3540 (87.0%) |
| *Missing* | *0 (0%)* | *0 (0%)* | *-* | *-* |
| **Gender²** |  |  |  |  |
| Female | 3267 (59.3%) | 2825 (58.7%) | 2741 (58.7%) | 2409 (59.2%) |
| Male | 2239 (40.7%) | 1991 (41.3%) | 1928 (41.3%) | 1659 (40.8%) |
| **Years of education** |  |  |  |  |
| Mean (SD) | 12.8 (2.89) | 13.0 (2.78) | 13.0 (2.75) | 13.2 (2.52) |
| Median [Q1-Q3] | 12.0 [12.0-15.0] | 12.0 [12.0-15.0] | 12.0 [12.0-15.0] | 12.0 [12.0-16.0] |
| *Missing* | *2 (0.0%)* | *2 (0.0%)* | *-* | *-* |
| **Born in a Southern US state** |  |  |  |  |
| Yes | 1755 (31.9%) | 1496 (31.1%) | 1440 (30.8%) | 1285 (31.6%) |
| No | 3746 (68.0%) | 3318 (68.9%) | 3229 (69.2%) | 2783 (68.4%) |
| *Missing* | *5 (0.1%)* | *2 (0.0%)* | *-* | *-* |
| **Parental years of education³** |  |  |  |  |
| <8 years | 877 (15.9%) | 730 (15.2%) | 698 (14.9%) | 511 (12.6%) |
| 8-12 years | 3296 (59.9%) | 2926 (60.8%) | 2854 (61.1%) | 2564 (63.0%) |
| >12 years | 1024 (18.6%) | 913 (19.0%) | 895 (19.2%) | 817 (20.1%) |
| Unknown | 309 (5.6%) | 247 (5.1%) | 222 (4.8%) | 176 (4.3%) |
| **Marital status** |  |  |  |  |
| Married/partnered | 3556 (64.6%) | 3176 (65.9%) | 3068 (65.7%) | 2686 (66.0%) |
| Separated/divorced/widowed | 1810 (32.9%) | 1529 (31.7%) | 1495 (32.0%) | 1289 (31.7%) |
| Never married | 139 (2.5%) | 110 (2.3%) | 106 (2.3%) | 93 (2.3%) |
| *Missing* | *1 (0.0%)* | *1 (0.0%)* | *-* | *-* |
| **Has employer-provided health insurance** |  |  |  |  |
| Yes | 1700 (30.9%) | 1540 (32.0%) | 1505 (32.2%) | 1363 (33.5%) |
| No | 3764 (68.4%) | 3242 (67.3%) | 3164 (67.8%) | 2705 (66.5%) |
| *Missing* | *42 (0.8%)* | *34 (0.7%)* | *-* | *-* |
| **Occupational skill level** |  |  |  |  |
| Higher skill | 361 (6.6%) | 330 (6.9%) | 321 (6.9%) | 301 (7.4%) |
| Lower skill | 660 (12.0%) | 590 (12.3%) | 575 (12.3%) | 511 (12.6%) |
| Not working | 4447 (80.8%) | 3861 (80.2%) | 3740 (80.1%) | 3225 (79.3%) |
| Unknown | 38 (0.7%) | 35 (0.7%) | 33 (0.7%) | 31 (0.8%) |
| **Wealth at baseline (in 2010 dollars)⁴** |  |  |  |  |
| Mean (SD) | 509000 (809000) | 528000 (827000) | 530000 (820000) | 552000 (832000) |
| Median [Q1-Q3] | 240000 [77900-615000] | 259000 [85800-644000] | 262000 [86700-649000] | 281000 [96800-688000] |
| **Income at baseline (in 2010 dollars)⁵** |  |  |  |  |
| Mean (SD) | 56300 (75600) | 58500 (77700) | 58600 (78000) | 61100 (81400) |
| Median [Q1-Q3] | 37900 [21900-65300] | 39300 [23300-67800] | 39400 [23300-68000] | 40700 [24500-70200] |
| **Drinking status** |  |  |  |  |
| Ever drinks alcohol | 2934 (53.3%) | 2613 (54.3%) | 2542 (54.4%) | 2237 (55.0%) |
| Never drinks alcohol | 2572 (46.7%) | 2203 (45.7%) | 2127 (45.6%) | 1831 (45.0%) |
| **Smoking status** |  |  |  |  |
| Currently smokes cigarettes | 412 (7.5%) | 360 (7.5%) | 350 (7.5%) | 280 (6.9%) |
| Never/formerly smoked cigarettes | 5056 (91.8%) | 4425 (91.9%) | 4319 (92.5%) | 3788 (93.1%) |
| *Missing* | *38 (0.7%)* | *31 (0.6%)* | *-* | *-* |
| **Body Mass Index** |  |  |  |  |
| Mean (SD) | 28.2 (5.58) | 28.3 (5.61) | 28.3 (5.61) | 28.4 (5.64) |
| Median [Q1-Q3] | 27.5 [24.4-31.2] | 27.5 [24.4-31.2] | 27.5 [24.4-31.2] | 27.5 [24.4-31.3] |
| *Missing* | *45 (0.8%)* | *40 (0.8%)* | *-* | *-* |
| **No. of doctor diagnosed health conditions** |  |  |  |  |
| Mean (SD) | 2.33 (1.35) | 2.30 (1.34) | 2.30 (1.33) | 2.28 (1.32) |
| Median [Q1-Q3] | 2.00 [1.00-3.00] | 2.00 [1.00-3.00] | 2.00 [1.00-3.00] | 2.00 [1.00-3.00] |
| **Depressive symptoms (CESD-8 score ≥4)** |  |  |  |  |
| Yes | 532 (9.7%) | 439 (9.1%) | 422 (9.0%) | 344 (8.5%) |
| No | 4929 (89.5%) | 4337 (90.1%) | 4247 (91.0%) | 3724 (91.5%) |
| *Missing* | *45 (0.8%)* | *40 (0.8%)* | *-* | *-* |
| **Composite memory z-score at baseline** |  |  |  |  |
| Mean (SD) | 0.00341 (0.816) | -0.000837 (0.817) | 0.0161 (0.798) | 0.037 (0.78) |
| Median [Q1-Q3] | 0.153 [-0.418-0.568] | 0.143 [-0.408-0.568] | 0.151 [-0.388-0.573] | 0.17 [-0.37-0.59] |
| *Missing* | *386 (7.0%)* | *308 (6.4%)* | 290 (6.2%) | *18 (0.4%)* |

**Appendix S3**: Results of validation analyses for financial well-being index

The sample for validation analyses comprised 59% female, 18% Black, 13% Hispanic, and 69% non-Black and non-Hispanic participants with a median [IQR] age of 68 [16] and years of education of 13 [4]. The sample median [IQR] FWB index score was 1 [2] and for CFPB-FWB was 62 [17]. As expected, FWB index scores and CFPB-FWB scores were inversely correlated (**Table S3a)**. The proportion of the sample in the highest CFPB-FWB quintiles decreased as FWB index scores increased (and vice versa); 19.3% of the sample had a FWB index score ≥3 (corresponding to a CFPB-FWB score of 52), a threshold that seemed to reasonably divide the sample into worse vs. better financial well-being. The Spearman correlation between FWB index and CFPB-FWB scores exceeded our accepted threshold of 0.50 for convergent validity ($\text{ρ}$=-0.65, 95% CI: -0.67, -0.63; **Figure S2**).

Correlations between FWB index scores and financial literacy measures were noticeably lower than for correlations with CFPB-FWB, providing evidence of discriminant validity. Specifically, Spearman correlations between summary scores for our poor FWB index and correct responses on financial literacy questions were -0.072 (95% CI: -0.177, 0.035) for understanding of interest compounding, -0.171 (95% CI: -0.272, -0.066) for understanding of inflation, and -0.145 (95% CI: -0.248, -0.040) for understanding of risk diversification. The correlation between FWB index summary scores and demonstrating financial literacy (i.e., getting all questions correct) was -0.168 (95% CI: -0.270, -0.063). All correlations were noticeably weaker than those between FWB index and CFPB-FWB scores (convergent validity analysis, $\text{ρ}$=-0.65), providing evidence of discriminant validity of our poor FWB index.

Our poor FWB index performed similarly in these discriminant validity analyses to the CFPB-FWB scale. For comparison, correlations between summary scores on the CFPB-FWB scale and correct responses on financial literacy questions were 0.043 (95% CI: -0.064, 0.149) for understanding of interest compounding, 0.170 (95% CI: 0.065, 0.272) for understanding of inflation, and 0.152 (95% CI: 0.047, 0.254) for understanding of risk diversification. The correlation between CFPB-FWB summary scores and demonstrating financial literacy was 0.185 (95% CI: 0.080, 0.285).

Internal consistency of the FWB index items was good (Cronbach’s alpha=0.702). Most CFPB-FWB items had at least one analog in our FWB index (**Figure S2**). Financial dissatisfaction ($\text{ρ}$=-0.52), ongoing financial strain ($\text{ρ}$=-0.51), and low non-housing wealth ($\text{ρ}$=-0.44) had the strongest correlations with CFPB-FWB scores, and median CFPB-FWB scores were lowest for individuals reporting financial dissatisfaction (48), difficulty paying bills (44), or ongoing financial strain (48, **Table S3b**), suggesting these may be particularly important items to include in a FWB index.

Finally, higher FWB index scores (indicating worse financial well-being) associated with a higher odds of fair/poor self-rated health (OR=1.55, 95% CI: 1.48, 1.62), receiving Medicaid (OR=1.84, 95% CI: 1.74, 1.95), receiving income from government assistance (OR=1.48, 95% CI: 1.41, 1.55), and reporting food insecurity (OR=2.20, 95% CI: 2.02, 2.40) (**Table S3c, row 1**). The magnitude of associations between z-scores for both financial well-being measures and each validation outcome were quite comparable (**Table S3c, rows 2 and 3**).

**Conclusions**

Our FWB index, constructed from 8 commonly used subjective and objective indicators of financial status [22, 23] that were serially collected in the HRS from 2004-2020, showed good internal consistency and was well-correlated with the CFPB-FWB scale. Z-scores on both FWB measures were strongly associated at similar magnitudes with all four validation outcomes, especially those strongly tied to financial hardship (*e.g.,* receipt of Medicaid, food insecurity). This suggests our index may adequately measure financial well-being in HRS survey years when the CFPB-FWB scale is unavailable. For many HRS participants, FWB index indicators are collected longitudinally, enabling evaluation of persistent/long-term exposure to poor financial well-being and its impact on later health. Further, other US cohort studies (*e.g.,* Midlife in the United States (MIDUS), [24]) have collected data on key items in our index like difficulty paying bills, money stress, assets/wealth, and financial satisfaction (albeit, often using different questions than those evaluated herein), suggesting at least a reduced version of this index could be constructed in other cohorts.

Financial dissatisfaction, difficulty paying bills, ongoing financial strain, and low non-housing wealth were key items in the index. In a sensitivity analysis including only these items, results were similar to those from the 8-item index (correlation with CFPB-FWB was -0.66 and Cronbach’s alpha=0.65). While a reduced index comprising these key indicators may sufficiently capture financial well-being, Cronbach’s alpha was slightly better, exceeding 0.70, when all 8 items were included. Still, as other studies may lack information on all 8 items, it helps to know a reduced index of key items may perform reasonably well as a measure of financial well-being. The item “taking less medication because of money” had fairly low correlations with all CFPB-FWB items, suggesting it could be dropped from the scale without altering performance of the index. However, we decided to retain it on theoretical grounds [25-27].

It is unclear if these findings generalize outside of the HRS context (adults aged >50 approaching/having entered retirement), so future studies should evaluate this index in other cohorts. Additionally, our findings may be specific to the 2020 HRS sample; however, all FWB index items and the CFPB-FWB scale were collected in the 2022 HRS survey, providing opportunity to replicate this analysis in the future.

**Table S3a.** Summary statistics for FWB index scores and CFPB-FWB scale scores in the 2020 HRS survey

| **FWB**  **index score** | **N** | **CFPB-FWB score**  **Med (25%, 75%)** | **CFPB-FWB score quintiles**  **row %** | | | | |
| --- | --- | --- | --- | --- | --- | --- | --- |
|  |  |  | **[14, 52)** | **[52, 58)** | **[58, 66)** | **[66, 75)** | **[75, 95]** |
| 0 | 1972 | 69 (62, 78) | 2% | 9% | 25% | 28% | 36% |
| 1 | 751 | 61 (55, 69) | 11% | 26% | 30% | 19% | 15% |
| 2 | 515 | 56 (51, 62) | 27% | 30% | 26% | 11% | 5% |
| 3 | 338 | 52 (46, 58) | 46% | 28% | 19% | 6% | 1% |
| 4 | 218 | 49 (44, 53) | 62% | 28% | 7% | 3% | 0% |
| 5 | 126 | 45 (39, 50) | 79% | 17% | 4% | 0% | 0% |
| 6 | 64 | 42 (37, 48) | 85% | 11% | 5% | 0% | 0% |
| 7 | 23 | 35 (29, 42) | 96% | 4% | 0% | 0% | 0% |
| 8 | 4 | 33 (29, 37) | 100% | 0% | 0% | 0% | 0% |

**Table S3b**. Median (25^th^%, 75^th^%) CFPB-FWB scores within levels of each binary FWB index item

|  | **CFPB-FWB score**  **Median (25^th^%, 75^th^%)** | |
| --- | --- | --- |
| **Financial indicators in FWB index** | **Yes**  **(FWB indicator=1)** | **No**  **(FWB indicator=0)** |
| Low control over finances | 50 (42, 57) | 63 (56, 73) |
| Low financial satisfaction | 48 (42, 53) | 64 (57, 75) |
| Difficulty paying bills | 44 (37, 49) | 63 (55, 73) |
| Ongoing financial strain that results in distress | 48 (41, 52) | 64 (57, 75) |
| Forgoing medications because of cost | 52 (45, 60) | 63 (54, 73) |
| Low income | 54 (48, 62) | 64 (56, 74) |
| No housing wealth | 55 (48, 62) | 64 (56, 75) |
| Low non-housing wealth | 53 (46, 60) | 66 (58, 75) |

**Table S3c.** Bivariate associations between FWB index scores and both FWB index and CFPB-FWB z-scores and four validation outcomes in the 2020 Health and Retirement Study sample

|  | **Validation Outcomes**  **OR (95% CI)** | | | |
| --- | --- | --- | --- | --- |
| **FWB measure (min-max)** | **Fair/poor**  **self-rated health**  **(N=4009)** | **Receiving Medicaid at any time since last survey**  **(N=3990)** | **Receiving income from government assistance in past calendar year**  **(N=4011)^a^** | **Reporting food insecurity since last survey or in past 12 months**  **(N=2855)^b^** |
| FWB index scores (1-8) | 1.55 (1.48, 1.62) | 1.84 (1.74, 1.95) | 1.48 (1.41, 1.55) | 2.20 (2.02, 2.40) |
| FWB index z-scores (-0.76-4.21)^c^ | 2.02 (1.88, 2.17) | 2.65 (2.43, 2.90) | 1.87 (1.73, 2.02) | 3.53 (3.08, 4.07) |
| CFPB-FWB z-scores (-2.36-3.56)^d^ | 2.16 (1.98, 2.35) | 2.44 (2.19, 2.73) | 1.72 (1.56, 1.90) | 4.07 (3.38, 4.94) |

^a^There was complete data on receiving income from government assistance because we pulled this outcome from the RAND longitudinal file, which incorporates imputations for missing income measures.

^b^Most missing data on food insecurity is due to survey administration procedures (asking only the financial respondent these questions).

^c^Higher scores indicate worse financial well-being

^d^Reverse coded so that higher scores indicate worse financial well-being

**Figure S2**. Spearman correlations between FWB index items and summary scores (rows) and CFPB-FWB scale items and summary scores (columns)

**
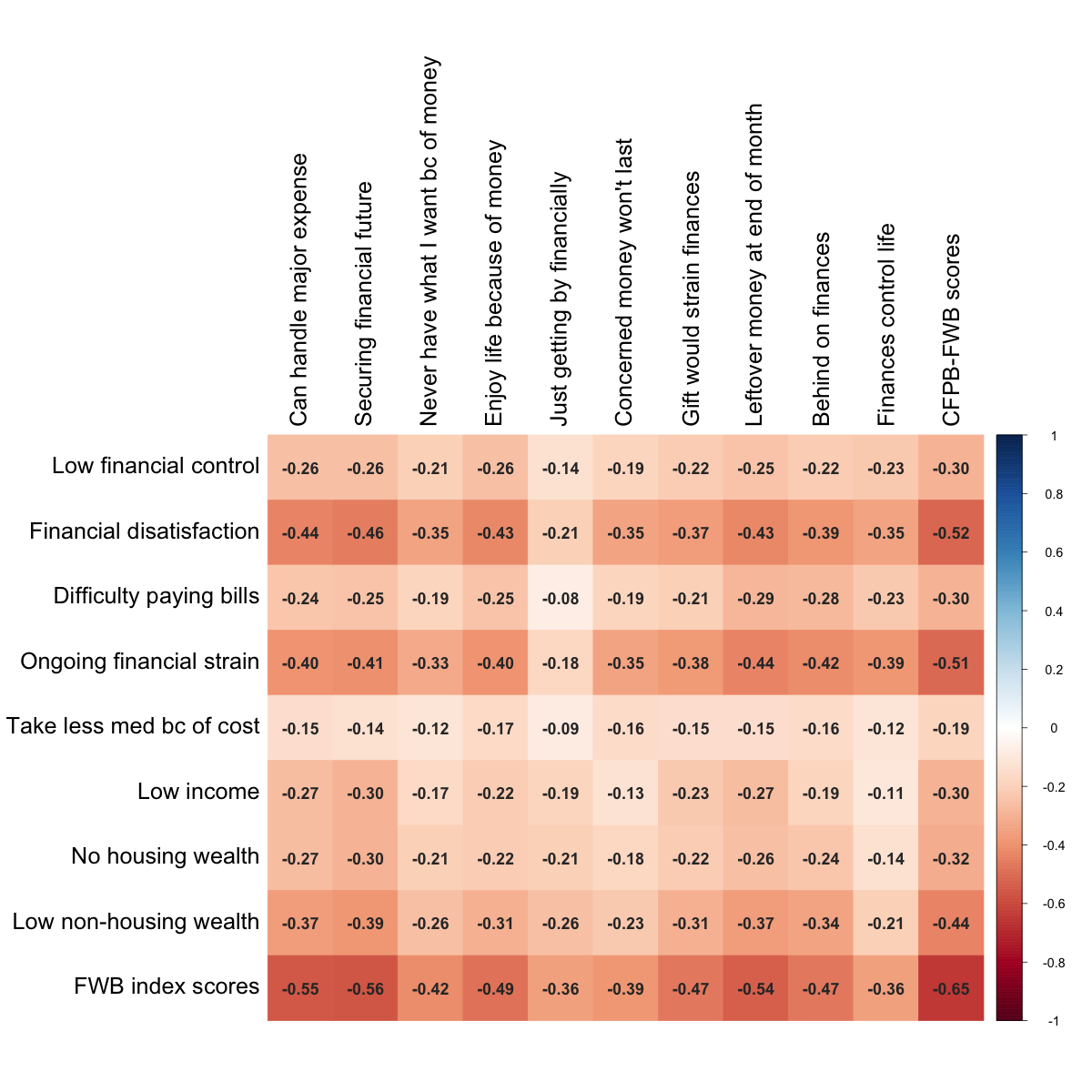
**

**Figure S2 Legend**

**Figure S2** shows a correlation plot between items and summary scores on our index of poor financial well-being (rows) and those on the Consumer Financial Protection Bureau’s Financial Well-Being Scale (columns).

**Appendix S4**: Regression and sensitivity analysis results

**Table S4a**. Confounder-adjusted associations between financial well-being exposures and memory function z-scores in 2016 and memory decline from 2016-2020 from sequentially adjusted models.

|  |  | **Model 1**  $\text{β}$ **(95% CI)** | **Model 2**  $\text{β}$ **(95% CI)** | **Model 3**  $\text{β}$ **(95% CI)** | **Model 4**  $\text{β}$ **(95% CI)** |
| --- | --- | --- | --- | --- | --- |
| **Total Sample** |  |  |  |  |  |
| Average FWB score |  | -0.0819 (-0.0921, -0.0716) | -0.0178 (-0.0274, -0.0082) | -0.0160 (-0.0267, -0.0052) | -0.0086 (-0.0199, 0.0026) |
| Time |  | -0.0438 (-0.0517, -0.0359) | -0.0440 (-0.0541, -0.0338) | -0.0439 (-0.0541, -0.0338) | -0.0440 (-0.0542, -0.0339) |
| Average FWB score*time |  | -0.0075 (-0.0107, -0.0044) | -0.0066 (-0.0099, -0.0033) | -0.0066 (-0.0099, -0.0033) | -0.0066 (-0.0099, -0.0033) |
| FWB score significantly improved |  | -0.0597 (-0.1123, -0.0071) | 0.0164 (-0.0288, 0.0616) | 0.0295 (-0.0163, 0.0752) | 0.0415 (-0.0044, 0.0873) |
| Time |  | -0.0517 (-0.0588, -0.0445) | -0.0536 (-0.0624, -0.0448) | -0.0536 (-0.0624, -0.0448) | -0.0537 (-0.0625, -0.0449) |
| FWB score significantly improved*time |  | -0.0117 (-0.0276, 0.0042) | -0.0092 (-0.0251, 0.0067) | -0.0091 (-0.0250, 0.0068) | -0.0091 (-0.0250, 0.0068) |
| FWB score significantly worsened |  | -0.1299 (-0.1934, -0.0664) | -0.0802 (-0.1348, -0.0256) | -0.0745 (-0.1292, -0.0198) | -0.0673 (-0.1221, -0.0126) |
| Time |  | -0.0511 (-0.0581, -0.0440) | -0.0526 (-0.0613, -0.0438) | -0.0526 (-0.0613, -0.0438) | -0.0527 (-0.0614, -0.0439) |
| FWB score significantly worsened*time |  | -0.0265 (-0.0459, -0.0070) | -0.0237 (-0.0432, -0.0042) | -0.0237 (-0.0432, -0.0042) | -0.0237 (-0.0432, -0.0042) |
| **Participants aged <65 years** |  |  |  |  |  |
| Average FWB score |  | -0.0739 (-0.0826, -0.0652) | -0.0124 (-0.0196, -0.0052) | -0.0047 (-0.0128, 0.0035) | 0.0011 (-0.0076, 0.0097) |
| Time |  | -0.0240 (-0.0404, -0.0077) | -0.0209 (-0.0380, -0.0039) | -0.0211 (-0.0382, -0.0041) | -0.0214 (-0.0384, -0.0044) |
| Average FWB score*time |  | -0.0069 (-0.0094, -0.0044) | -0.0058 (-0.0084, -0.0032) | -0.0058 (-0.0084, -0.0032) | -0.0058 (-0.0084, -0.0032) |
| FWB score significantly improved |  | -0.0488 (-0.0926, -0.0050) | 0.0168 (-0.0147, 0.0483) | 0.0325 ( 0.0006, 0.0644) | 0.0376 ( 0.0057, 0.0696) |
| Time |  | -0.0290 (-0.0453, -0.0127) | -0.0280 (-0.0447, -0.0113) | -0.0283 (-0.0451, -0.0116) | -0.0286 (-0.0453, -0.0119) |
| FWB score significantly improved*time |  | -0.0101 (-0.0219, 0.0017) | -0.0079 (-0.0196, 0.0039) | -0.0077 (-0.0194, 0.0041) | -0.0076 (-0.0193, 0.0042) |
| FWB score significantly worsened |  | -0.0720 (-0.1332, -0.0108) | -0.0326 (-0.0765, 0.0114) | -0.0283 (-0.0723, 0.0156) | -0.0216 (-0.0657, 0.0224) |
| Time |  | -0.0298 (-0.0461, -0.0135) | -0.0287 (-0.0454, -0.0120) | -0.0290 (-0.0457, -0.0123) | -0.0292 (-0.0459, -0.0125) |
| FWB score significantly worsened*time |  | -0.0065 (-0.0229, 0.0099) | -0.0039 (-0.0202, 0.0124) | -0.0039 (-0.0202, 0.0124) | -0.0040 (-0.0203, 0.0123) |
| **Participants aged ≥65 years** |  |  |  |  |  |
| Average FWB score |  | -0.0933 (-0.1131, -0.0735) | -0.0371 (-0.0557, -0.0184) | -0.0314 (-0.0519, -0.0109) | -0.0217 (-0.0428, -0.0005) |
| Time |  | -0.0520 (-0.0748, -0.0292) | -0.0539 (-0.0791, -0.0287) | -0.0538 (-0.0790, -0.0285) | -0.0539 (-0.0792, -0.0287) |
| Average FWB score*time |  | -0.0086 (-0.0150, -0.0021) | -0.0079 (-0.0146, -0.0012) | -0.0079 (-0.0146, -0.0013) | -0.0079 (-0.0146, -0.0013) |
| FWB score significantly improved |  | -0.0687 (-0.1780, 0.0406) | 0.0074 (-0.0893, 0.1041) | 0.0325 (-0.0651, 0.1302) | 0.0632 (-0.0351, 0.1614) |
| Time |  | -0.0594 (-0.0815, -0.0374) | -0.0637 (-0.0875, -0.0399) | -0.0636 (-0.0874, -0.0398) | -0.0638 (-0.0876, -0.0400) |
| FWB score significantly improved*time |  | -0.0160 (-0.0517, 0.0197) | -0.0118 (-0.0476, 0.0240) | -0.0118 (-0.0476, 0.0240) | -0.0117 (-0.0475, 0.0241) |
| FWB score significantly worsened |  | -0.1852 (-0.2933, -0.0770) | -0.1292 (-0.2252, -0.0333) | -0.1200 (-0.2163, -0.0237) | -0.1109 (-0.2073, -0.0145) |
| Time |  | -0.0573 (-0.0792, -0.0354) | -0.0606 (-0.0842, -0.0369) | -0.0605 (-0.0841, -0.0368) | -0.0606 (-0.0843, -0.0370) |
| FWB score significantly worsened*time |  | -0.0490 (-0.0848, -0.0131) | -0.0463 (-0.0823, -0.0103) | -0.0461 (-0.0821, -0.0101) | -0.0464 (-0.0824, -0.0104) |

Model 1 included adjustment for age (centered at 65 years), age-squared, and their interactions with time.

Model 2 adjusted for Model 1 covariates plus race, sex/gender and its interaction with time, years of education (centered at 12) and its interaction with time, being born in the South, and parental educational attainment.

Model 3 adjusted for Model 2 covariates plus marital status, having employer-provided health insurance, occupational skill level/working status, total household wealth, and total household income (log transformed after adding a small correction factor of 0.001).

Model 4 adjusted for Model 3 covariates plus alcohol drinking status, smoking status, BMI (mean-centered), number of chronic health conditions, and depressive symptomatology.

**Table S4b**. Confounder-adjusted associations between financial well-being exposures and memory function z-scores in 2016 and memory decline from 2016-2020 from sequentially adjusted models: sensitivity analyses addressing reverse causation by removing all participants with poor memory function at study baseline (2010/2012).

|  |  | **Model 1**  $\text{β}$ **(95% CI)** | **Model 2**  $\text{β}$ **(95% CI)** | **Model 3**  $\text{β}$ **(95% CI)** | **Model 4**  $\text{β}$ **(95% CI)** |
| --- | --- | --- | --- | --- | --- |
| **Total Sample** |  |  |  |  |  |
| Average FWB score |  | -0.0727 (-0.0825, -0.0629) | -0.0168 (-0.0261, -0.0075) | -0.0134 (-0.0238, -0.0030) | -0.0058 (-0.0167, 0.0050) |
| Time |  | -0.0433 (-0.0512, -0.0353) | -0.0437 (-0.0539, -0.0336) | -0.0437 (-0.0539, -0.0336) | -0.0439 (-0.0540, -0.0337) |
| Average FWB score*time |  | -0.0071 (-0.0103, -0.0040) | -0.0061 (-0.0094, -0.0028) | -0.0061 (-0.0094, -0.0028) | -0.0061 (-0.0095, -0.0028) |
| FWB score significantly improved |  | -0.0576 (-0.1075, -0.0077) | 0.0117 (-0.0317, 0.0551) | 0.0253 (-0.0186, 0.0692) | 0.0373 (-0.0067, 0.0814) |
| Time |  | -0.0506 (-0.0578, -0.0434) | -0.0525 (-0.0613, -0.0436) | -0.0525 (-0.0614, -0.0436) | -0.0526 (-0.0615, -0.0438) |
| FWB score significantly improved*time |  | -0.0115 (-0.0273, 0.0042) | -0.0091 (-0.0248, 0.0067) | -0.0090 (-0.0248, 0.0068) | -0.0090 (-0.0248, 0.0068) |
| FWB score significantly worsened |  | -0.1049 (-0.1665, -0.0433) | -0.0696 (-0.1232, -0.0160) | -0.0639 (-0.1176, -0.0102) | -0.0562 (-0.1099, -0.0024) |
| Time |  | -0.0506 (-0.0577, -0.0434) | -0.0522 (-0.0610, -0.0434) | -0.0522 (-0.0610, -0.0434) | -0.0523 (-0.0611, -0.0435) |
| FWB score significantly worsened*time |  | -0.0185 (-0.0381, 0.0012) | -0.0160 (-0.0356, 0.0037) | -0.0159 (-0.0356, 0.0038) | -0.0159 (-0.0356, 0.0037) |
| **Participants aged <65 years** |  |  |  |  |  |
| Average FWB score |  | -0.0735 (-0.0822, -0.0648) | -0.0125 (-0.0196, -0.0053) | -0.0046 (-0.0128, 0.0035) | 0.0013 (-0.0073, 0.0099) |
| Time |  | -0.0228 (-0.0391, -0.0065) | -0.0204 (-0.0374, -0.0034) | -0.0206 (-0.0376, -0.0036) | -0.0209 (-0.0379, -0.0038) |
| Average FWB score*time |  | -0.0068 (-0.0093, -0.0044) | -0.0057 (-0.0084, -0.0031) | -0.0057 (-0.0083, -0.0031) | -0.0057 (-0.0083, -0.0031) |
| FWB score significantly improved |  | -0.0497 (-0.0934, -0.0060) | 0.0161 (-0.0154, 0.0476) | 0.0318 ( 0.0000, 0.0636) | 0.0371 ( 0.0052, 0.0689) |
| Time |  | -0.0276 (-0.0439, -0.0113) | -0.0273 (-0.0440, -0.0105) | -0.0276 (-0.0443, -0.0109) | -0.0279 (-0.0446, -0.0111) |
| FWB score significantly improved*time |  | -0.0102 (-0.0220, 0.0015) | -0.0080 (-0.0197, 0.0038) | -0.0078 (-0.0195, 0.0040) | -0.0077 (-0.0194, 0.0041) |
| FWB score significantly worsened |  | -0.0722 (-0.1334, -0.0110) | -0.0326 (-0.0766, 0.0113) | -0.0283 (-0.0723, 0.0156) | -0.0214 (-0.0654, 0.0226) |
| Time |  | -0.0284 (-0.0447, -0.0121) | -0.0280 (-0.0447, -0.0112) | -0.0282 (-0.0450, -0.0115) | -0.0285 (-0.0452, -0.0117) |
| FWB score significantly worsened*time |  | -0.0074 (-0.0237, 0.0090) | -0.0048 (-0.0211, 0.0115) | -0.0047 (-0.0210, 0.0116) | -0.0048 (-0.0211, 0.0115) |
| **Participants aged ≥65 years** |  |  |  |  |  |
| Average FWB score |  | -0.0704 (-0.0899, -0.0508) | -0.0317 (-0.0504, -0.0131) | -0.0264 (-0.0469, -0.0058) | -0.0164 (-0.0376, 0.0048) |
| Time |  | -0.0531 (-0.0763, -0.0299) | -0.0561 (-0.0817, -0.0304) | -0.0560 (-0.0816, -0.0303) | -0.0561 (-0.0817, -0.0304) |
| Average FWB score*time |  | -0.0076 (-0.0142, -0.0009) | -0.0068 (-0.0137, 0.0000) | -0.0069 (-0.0137, 0.0000) | -0.0068 (-0.0137, 0.0000) |
| FWB score significantly improved |  | -0.0652 (-0.1713, 0.0409) | -0.0046 (-0.1012, 0.0920) | 0.0173 (-0.0802, 0.1148) | 0.0502 (-0.0481, 0.1485) |
| Time |  | -0.0597 (-0.0821, -0.0374) | -0.0642 (-0.0884, -0.0401) | -0.0642 (-0.0883, -0.0400) | -0.0643 (-0.0884, -0.0401) |
| FWB score significantly improved*time |  | -0.0155 (-0.0520, 0.0210) | -0.0118 (-0.0484, 0.0248) | -0.0119 (-0.0485, 0.0247) | -0.0118 (-0.0484, 0.0247) |
| FWB score significantly worsened |  | -0.1411 (-0.2495, -0.0328) | -0.1164 (-0.2151, -0.0177) | -0.1083 (-0.2074, -0.0092) | -0.0977 (-0.1970, 0.0016) |
| Time |  | -0.0587 (-0.0810, -0.0365) | -0.0626 (-0.0866, -0.0385) | -0.0625 (-0.0866, -0.0385) | -0.0626 (-0.0867, -0.0386) |
| FWB score significantly worsened*time |  | -0.0320 (-0.0696, 0.0057) | -0.0301 (-0.0679, 0.0077) | -0.0299 (-0.0677, 0.0079) | -0.0300 (-0.0678, 0.0078) |

Model 1 included adjustment for age (centered at 65 years), age-squared, and their interactions with time.

Model 2 adjusted for Model 1 covariates plus race, sex/gender and its interaction with time, years of education (centered at 12) and its interaction with time, being born in the South, and parental educational attainment.

Model 3 adjusted for Model 2 covariates plus marital status, having employer-provided health insurance, occupational skill level/working status, total household wealth, and total household income (log transformed after adding a small correction factor of 0.001).

Model 4 adjusted for Model 3 covariates plus alcohol drinking status, smoking status, BMI (mean-centered), number of chronic health conditions, and depressive symptomatology.

**Table S4c**. Confounder-adjusted associations between financial well-being exposures and memory function z-scores in 2016 and memory decline from 2016-2020 from sequentially adjusted models: sensitivity analyses using changes in memory function from study baseline as outcomes in 2016-2020.

|  |  | **Model 1**  $\text{β}$ **(95% CI)** | **Model 2**  $\text{β}$ **(95% CI)** | **Model 3**  $\text{β}$ **(95% CI)** | **Model 4**  $\text{β}$ **(95% CI)** |
| --- | --- | --- | --- | --- | --- |
| **Total Sample** |  |  |  |  |  |
| Average FWB score |  | -0.0355 (-0.0485, -0.0224) | -0.0195 (-0.0336, -0.0055) | -0.0154 (-0.0311, 0.0003) | -0.0098 (-0.0263, 0.0067) |
| Time |  | -0.0651 (-0.0772, -0.0529) | -0.0654 (-0.0809, -0.0500) | -0.0655 (-0.0809, -0.0500) | -0.0655 (-0.0810, -0.0501) |
| Average FWB score*time |  | -0.0117 (-0.0165, -0.0068) | -0.0103 (-0.0154, -0.0052) | -0.0103 (-0.0154, -0.0052) | -0.0103 (-0.0154, -0.0053) |
| FWB score improved by ≥2 points |  | 0.0061 (-0.0600, 0.0721) | 0.0286 (-0.0374, 0.0946) | 0.0462 (-0.0206, 0.1130) | 0.0571 (-0.0100, 0.1241) |
| Time |  | -0.0775 (-0.0885, -0.0666) | -0.0805 (-0.0939, -0.0670) | -0.0805 (-0.0940, -0.0671) | -0.0806 (-0.0941, -0.0672) |
| FWB score improved by ≥2 points*time |  | -0.0165 (-0.0409, 0.0079) | -0.0139 (-0.0383, 0.0104) | -0.0138 (-0.0382, 0.0105) | -0.0139 (-0.0382, 0.0105) |
| FWB score worsened by ≥2 points |  | -0.0457 (-0.1255, 0.0341) | -0.0223 (-0.1021, 0.0575) | -0.0194 (-0.0995, 0.0606) | -0.0142 (-0.0944, 0.0660) |
| Time |  | -0.0764 (-0.0872, -0.0656) | -0.0787 (-0.0921, -0.0653) | -0.0788 (-0.0921, -0.0654) | -0.0789 (-0.0922, -0.0655) |
| FWB score worsened by ≥2 points*time |  | -0.0412 (-0.0710, -0.0114) | -0.0384 (-0.0683, -0.0086) | -0.0384 (-0.0682, -0.0086) | -0.0385 (-0.0683, -0.0087) |
| **Participants aged <65 years** |  |  |  |  |  |
| Average FWB score |  | -0.0322 (-0.0437, -0.0208) | -0.0071 (-0.0196, 0.0054) | 0.0064 (-0.0081, 0.0208) | 0.0096 (-0.0057, 0.0249) |
| Time |  | -0.0336 (-0.0586, -0.0086) | -0.0331 (-0.0592, -0.0070) | -0.0334 (-0.0594, -0.0073) | -0.0334 (-0.0595, -0.0073) |
| Average FWB score*time |  | -0.0104 (-0.0142, -0.0066) | -0.0085 (-0.0125, -0.0045) | -0.0085 (-0.0125, -0.0045) | -0.0085 (-0.0125, -0.0045) |
| FWB score improved by ≥2 points |  | 0.0053 (-0.0502, 0.0608) | 0.0336 (-0.0212, 0.0883) | 0.0543 (-0.0013, 0.1098) | 0.0570 ( 0.0014, 0.1127) |
| Time |  | -0.0414 (-0.0663, -0.0165) | -0.0435 (-0.0691, -0.0179) | -0.0438 (-0.0694, -0.0182) | -0.0439 (-0.0695, -0.0183) |
| FWB score improved by ≥2 points*time |  | -0.0147 (-0.0327, 0.0034) | -0.0114 (-0.0294, 0.0066) | -0.0112 (-0.0292, 0.0068) | -0.0112 (-0.0292, 0.0067) |
| FWB score worsened by ≥2 points |  | -0.0109 (-0.0885, 0.0668) | 0.0182 (-0.0584, 0.0948) | 0.0218 (-0.0549, 0.0984) | 0.0244 (-0.0525, 0.1012) |
| Time |  | -0.0423 (-0.0672, -0.0174) | -0.0442 (-0.0698, -0.0186) | -0.0445 (-0.0701, -0.0189) | -0.0446 (-0.0701, -0.0190) |
| FWB score worsened by ≥2 points*time |  | -0.0122 (-0.0373, 0.0129) | -0.0091 (-0.0341, 0.0159) | -0.0091 (-0.0341, 0.0159) | -0.0091 (-0.0340, 0.0159) |
| **Participants aged ≥65 years** |  |  |  |  |  |
| Average FWB score |  | -0.0411 (-0.0661, -0.0161) | -0.0332 (-0.0596, -0.0067) | -0.0367 (-0.0658, -0.0076) | -0.0299 (-0.0600, 0.0002) |
| Time |  | -0.0799 (-0.1148, -0.0450) | -0.0823 (-0.1209, -0.0437) | -0.0821 (-0.1207, -0.0435) | -0.0823 (-0.1209, -0.0437) |
| Average FWB score*time |  | -0.0135 (-0.0233, -0.0037) | -0.0125 (-0.0227, -0.0023) | -0.0126 (-0.0227, -0.0024) | -0.0126 (-0.0228, -0.0024) |
| FWB score improved by ≥2 points |  | 0.0100 (-0.1272, 0.1471) | 0.0260 (-0.1115, 0.1635) | 0.0374 (-0.1016, 0.1764) | 0.0638 (-0.0764, 0.2040) |
| Time |  | -0.0924 (-0.1261, -0.0586) | -0.0978 (-0.1342, -0.0614) | -0.0976 (-0.1340, -0.0612) | -0.0978 (-0.1342, -0.0614) |
| FWB score improved by ≥2 points*time |  | -0.0209 (-0.0757, 0.0338) | -0.0185 (-0.0733, 0.0364) | -0.0185 (-0.0734, 0.0363) | -0.0186 (-0.0734, 0.0363) |
| FWB score worsened by ≥2 points |  | -0.0788 (-0.2147, 0.0571) | -0.0598 (-0.1963, 0.0766) | -0.0559 (-0.1930, 0.0812) | -0.0490 (-0.1866, 0.0886) |
| Time |  | -0.0889 (-0.1224, -0.0554) | -0.0930 (-0.1292, -0.0568) | -0.0929 (-0.1291, -0.0567) | -0.0931 (-0.1293, -0.0569) |
| FWB score worsened by ≥2 points*time |  | -0.0734 (-0.1282, -0.0186) | -0.0710 (-0.1260, -0.0160) | -0.0710 (-0.1260, -0.0160) | -0.0713 (-0.1263, -0.0163) |

Model 1 included adjustment for age (centered at 65 years), age-squared, and their interactions with time.

Model 2 adjusted for Model 1 covariates plus race, sex/gender and its interaction with time, years of education (centered at 12) and its interaction with time, being born in the South, and parental educational attainment.

Model 3 adjusted for Model 2 covariates plus marital status, having employer-provided health insurance, occupational skill level/working status, total household wealth, and total household income (log transformed after adding a small correction factor of 0.001).

Model 4 adjusted for Model 3 covariates plus alcohol drinking status, smoking status, BMI (mean-centered), number of chronic health conditions, and depressive symptomatology.

**Table S4d**. Confounder-adjusted associations between financial well-being exposures and memory function z-scores in 2016 and memory decline from 2016-2020: sensitivity analysis additionally adjusting Model 4 (fully confounder-adjusted) for changes in marital status, employment status, BMI, and health status from exposure baseline (2010/2012) to exposure follow-up (2014/2016).

|  | **N** | **Model 4**  $\text{β}$ **(95% CI)** | **Model 4s**  $\text{β}$ **(95% CI)** |
| --- | --- | --- | --- |
| **Total Sample** |  |  |  |
| Average FWB score |  | -0.0086 (-0.0199, 0.0026) | -0.0082 (-0.0195, 0.0032) |
| Time |  | -0.0440 (-0.0542, -0.0339) | -0.0420 (-0.0528, -0.0313) |
| Average FWB score*time |  | -0.0066 (-0.0099, -0.0033) | -0.0061 (-0.0094, -0.0027) |
| FWB score significantly improved |  | 0.0415 (-0.0044, 0.0873) | 0.0441 (-0.0020, 0.0901) |
| Time |  | -0.0537 (-0.0625, -0.0449) | -0.0501 (-0.0597, -0.0404) |
| FWB score significantly improved*time |  | -0.0091 (-0.0250, 0.0068) | -0.0087 (-0.0247, 0.0074) |
| FWB score significantly worsened |  | -0.0673 (-0.1221, -0.0126) | -0.0667 (-0.1219, -0.0115) |
| Time |  | -0.0527 (-0.0614, -0.0439) | -0.0493 (-0.0589, -0.0397) |
| FWB score significantly worsened*time |  | -0.0237 (-0.0432, -0.0042) | -0.0238 (-0.0434, -0.0041) |
| **Participants aged <65 years** |  |  |  |
| Average FWB score |  | 0.0011 (-0.0076, 0.0097) | 0.0013 (-0.0074, 0.0100) |
| Time |  | -0.0214 (-0.0384, -0.0044) | -0.0245 (-0.0418, -0.0072) |
| Average FWB score*time |  | -0.0058 (-0.0084, -0.0032) | -0.0054 (-0.0081, -0.0028) |
| FWB score significantly improved |  | 0.0376 ( 0.0057, 0.0696) | 0.0379 ( 0.0059, 0.0700) |
| Time |  | -0.0286 (-0.0453, -0.0119) | -0.0303 (-0.0473, -0.0132) |
| FWB score significantly improved*time |  | -0.0076 (-0.0193, 0.0042) | -0.0074 (-0.0192, 0.0044) |
| FWB score significantly worsened |  | -0.0216 (-0.0657, 0.0224) | -0.0232 (-0.0677, 0.0213) |
| Time |  | -0.0292 (-0.0459, -0.0125) | -0.0310 (-0.0480, -0.0139) |
| FWB score significantly worsened*time |  | -0.0040 (-0.0203, 0.0123) | -0.0030 (-0.0195, 0.0135) |
| **Participants aged ≥65 years** |  |  |  |
| Average FWB score |  | -0.0217 (-0.0428, -0.0005) | -0.0214 (-0.0426, -0.0002) |
| Time |  | -0.0539 (-0.0792, -0.0287) | -0.0485 (-0.0747, -0.0223) |
| Average FWB score*time |  | -0.0079 (-0.0146, -0.0013) | -0.0076 (-0.0143, -0.0009) |
| FWB score significantly improved |  | 0.0632 (-0.0351, 0.1614) | 0.0712 (-0.0275, 0.1699) |
| Time |  | -0.0638 (-0.0876, -0.0400) | -0.0575 (-0.0825, -0.0326) |
| FWB score significantly improved*time |  | -0.0117 (-0.0475, 0.0241) | -0.0110 (-0.0470, 0.0251) |
| FWB score significantly worsened |  | -0.1109 (-0.2073, -0.0145) | -0.1102 (-0.2071, -0.0133) |
| Time |  | -0.0606 (-0.0843, -0.0370) | -0.0545 (-0.0792, -0.0297) |
| FWB score significantly worsened*time |  | -0.0464 (-0.0824, -0.0104) | -0.0481 (-0.0842, -0.0119) |

Model 4 included adjustment for age (centered at 65 years), age-squared, and their interactions with time, race, sex/gender and its interaction with time, years of education (centered at 12) and its interaction with time, being born in the South, and parental educational attainment, marital status, having employer-provided health insurance, occupational skill level/working status, total household wealth, and total household income (log transformed after adding a small correction factor of 0.001), alcohol drinking status, smoking status, BMI (mean-centered), number of chronic health conditions, and depressive symptomatology.

Model 4s includes Model 4 covariates plus indicators for experiencing the following over the exposure period: a marriage ending, becoming unemployed, significant weight loss, and a newly diagnosed health condition and each of their interactions with time.

**Table S4e**. Confounder-adjusted associations between financial well-being exposures and memory function z-scores in 2016 and memory decline from 2016-2020 from sequentially adjusted models: sensitivity analysis comparing unweighted Model 4 (original) with weighted Model 4 that accounts for attrition and missing data

|  |  | **Unweighted LMMs**  **(original model)** | **IP weighted LMMs**  **(using WeMix)^1^** |
| --- | --- | --- | --- |
|  |  | **Model 4^2^**  $\text{β}$ **(95% CI)** | **Model 4^2^**  $\text{β}$ **(95% CI)** |
| **Total Sample** |  |  |  |
| Average FWB score |  | -0.0086 (-0.0199, 0.0026) | -0.0117 (-0.0224, -0.0010) |
| Time |  | -0.0440 (-0.0542, -0.0339) | -0.0450 (-0.0560, -0.0340) |
| Average FWB score*time |  | -0.0066 (-0.0099, -0.0033) | -0.0069 (-0.0099, -0.0038) |
| FWB score significantly improved |  | 0.0415 (-0.0044, 0.0873) | 0.0399 ( 0.0010, 0.0789) |
| Time |  | -0.0537 (-0.0625, -0.0449) | -0.0551 (-0.0649, -0.0453) |
| FWB score significantly improved*time |  | -0.0091 (-0.0250, 0.0068) | -0.0098 (-0.0233, 0.0036) |
| FWB score significantly worsened |  | -0.0673 (-0.1221, -0.0126) | -0.0789 (-0.1426, -0.0152) |
| Time |  | -0.0527 (-0.0614, -0.0439) | -0.0539 (-0.0637, -0.0441) |
| FWB score significantly worsened*time |  | -0.0237 (-0.0432, -0.0042) | -0.0265 (-0.0486, -0.0044) |
| **Participants aged <65 years** |  |  |  |
| Average FWB score |  | 0.0011 (-0.0076, 0.0097) | 0.0007 (-0.0081, 0.0094) |
| Time |  | -0.0214 (-0.0384, -0.0044) | -0.0202 (-0.0417, 0.0013) |
| Average FWB score*time |  | -0.0058 (-0.0084, -0.0032) | -0.0059 (-0.0086, -0.0032) |
| FWB score significantly improved |  | 0.0376 ( 0.0057, 0.0696) | 0.0376 ( 0.0083, 0.0670) |
| Time |  | -0.0286 (-0.0453, -0.0119) | -0.0277 (-0.0485, -0.0069) |
| FWB score significantly improved*time |  | -0.0076 (-0.0193, 0.0042) | -0.0075 (-0.0200, 0.0049) |
| FWB score significantly worsened |  | -0.0216 (-0.0657, 0.0224) | -0.0211 (-0.0708, 0.0287) |
| Time |  | -0.0292 (-0.0459, -0.0125) | -0.0285 (-0.0494, -0.0075) |
| FWB score significantly worsened*time |  | -0.0040 (-0.0203, 0.0123) | -0.0023 (-0.0200, 0.0153) |
| **Participants aged ≥65 years** |  |  |  |
| Average FWB score |  | -0.0217 (-0.0428, -0.0005) | -0.0260 (-0.0458, -0.0062) |
| Time |  | -0.0539 (-0.0792, -0.0287) | -0.0540 (-0.0765, -0.0314) |
| Average FWB score*time |  | -0.0079 (-0.0146, -0.0013) | -0.0081 (-0.0145, -0.0018) |
| FWB score significantly improved |  | 0.0632 (-0.0351, 0.1614) | 0.0574 (-0.0349, 0.1496) |
| Time |  | -0.0638 (-0.0876, -0.0400) | -0.0641 (-0.0854, -0.0429) |
| FWB score significantly improved*time |  | -0.0117 (-0.0475, 0.0241) | -0.0136 (-0.0453, 0.0182) |
| FWB score significantly worsened |  | -0.1109 (-0.2073, -0.0145) | -0.1290 (-0.2385, -0.0195) |
| Time |  | -0.0606 (-0.0843, -0.0370) | -0.0608 (-0.0819, -0.0397) |
| FWB score significantly worsened*time |  | -0.0464 (-0.0824, -0.0104) | -0.0514 (-0.0928, -0.0101) |

^1^Models were weighted with stabilized weights for the inverse of one’s probability of being a complete case (among the full sample of N=10277) vs. not. Weighted linear mixed models were run using the WeMix package in R, using stabilized inverse probability weights at the participant level, with observation-level weights fixed at 1.

^2^Model 4 included adjustment for age (centered at 65 years), age-squared, and their interactions with time; race, sex/gender and its interaction with time, years of education (centered at 12) and its interaction with time, being born in the South, and parental educational attainment; marital status, having employer-provided health insurance, occupational skill level/working status, total household wealth, and total household income (log transformed after adding a small correction factor of 0.001) ; and alcohol drinking status, smoking status, BMI (mean-centered), number of chronic health conditions, and depressive symptomatology.

**Supplemental Data File References**

1. Sonnega, A., et al., *Cohort Profile: the Health and Retirement Study (HRS).* Int J Epidemiol, 2014. **43**(2): p. 576–85.

2. Clarke, P., et al., *Guide to content of the HRS psychosocial leave-behind participant lifestyle questionnaires: 2004 & 2006.* Ann Arbor, MI: University of Michigan, 2008.

3. Smith, J., et al., *Psychosocial and lifestyle questionnaire 2006-2022*, I.f.S.R. Survey Research Center, Editor. 2023, University of Michigan: Ann Arbor, Michigan.

4. RAND HRS Longitudinal File 2020 (V2). 2024: Produced by the RAND Center for the Study of Aging with funding from the National Institute on Aging and the Social Security Administration. Santa Monica, California.

5. Health and Retirement Study, *2020 HRS Final Core public use dataset.* 2020: Produced and distributed by the University of Michigan with funding from the National Institute on Aging (grant number NIA U01AG009740). Ann Arbor, Michigan.

6. Consumer Financial Protection Bureau. *CFPB financial well-being scale: scale development technical report*. 2017 12/19/2024]; Available from: <https://www.consumerfinance.gov/data-research/research-reports/financial-well-being-technical-report/>.

7. Consumer Financial Protection Bureau. *Measuring financial well-being: a guide to using the CFPB financial well-being scale*. 2015 9/18/2024]; Available from: <https://www.consumerfinance.gov/data-research/research-reports/financial-well-being-scale/>.

8. Lusardi, A. and O.S. Mitchell, *Financial literacy around the world: an overview.* Journal of pension economics & finance, 2011. **10**(4): p. 497–508.

9. Lusardi, A. and J.L. Streeter, *Financial literacy and financial well-being: Evidence from the US.* Journal of Financial Literacy and Wellbeing, 2023. **1**(2): p. 169–198.

10. Abma, I.L., M. Rovers, and P.J. van der Wees, *Appraising convergent validity of patient-reported outcome measures in systematic reviews: constructing hypotheses and interpreting outcomes.* BMC Res Notes, 2016. **9**: p. 226.

11. Warmath, D., *Financial literacy and financial well-being*, in *The Routledge handbook of financial literacy*. 2021, Routledge. p. 505–516.

12. Hubley, A.M., *Discriminant Validity*, in *Encyclopedia of Quality of Life and Well-Being Research*, A.C. Michalos, Editor. 2014, Springer Netherlands: Dordrecht. p. 1664–1667.

13. Boateng, G.O., et al., *Best Practices for Developing and Validating Scales for Health, Social, and Behavioral Research: A Primer.* Front Public Health, 2018. **6**: p. 149.

14. Gharaibeh, B., A.M. Al-Smadi, and D. Boyle, *Psychometric properties and characteristics of the Diabetes Self Management Scale.* Int J Nurs Sci, 2017. **4**(3): p. 252–259.

15. Taber, K.S., *The Use of Cronbach’s Alpha When Developing and Reporting Research Instruments in Science Education.* Research in Science Education, 2018. **48**(6): p. 1273–1296.

16. Tavakol, M. and R. Dennick, *Making sense of Cronbach's alpha.* Int J Med Educ, 2011. **2**: p. 53–55.

17. Streiner, D.L., et al., *77Selecting the items*, in *Health Measurement Scales: A practical guide to their development and use*. 2008, Oxford University Press. p. 0.

18. Bland, J.M. and D.G. Altman, *Statistics Notes: Validating scales and indexes.* Bmj, 2002. **324**(7337): p. 606–7.

19. Andri Signorell et mult. al. *DescTools: Tools for descriptive statistics.* 2017; Available from: <https://cran.r-project.org/package=DescTools>.

20. R Core Team, *A language and environment for statistical computing.* (No Title), 2021.

21. Rizopoulos, D. and M.D. Rizopoulos, *Package ‘ltm’.* URL <http://wiki>. r-project. org/rwiki/doku. php, 2018.

22. Samuel, L.J., et al., *Financial strain measures and associations with adult health: A systematic literature review.* Social Science & Medicine, 2025. **364**: p. 117531.

23. Glei, D.A., N. Goldman, and M. Weinstein, *Perception has its Own Reality: Subjective versus Objective Measures of Economic Distress.* Popul Dev Rev, 2018. **44**(4): p. 695–722.

24. Surachman, A., R. Tucker-Seeley, and D.M. Almeida, *The association between material-psychological-behavioral framework of financial hardship and markers of inflammation: a cross-sectional study of the Midlife in the United States (MIDUS) Refresher cohort.* BMC Public Health, 2023. **23**(1): p. 1845.

25. Walker, R.J., et al., *Relationship Between Multiple Measures of Financial Hardship and Glycemic Control in Older Adults With Diabetes.* J Appl Gerontol, 2021. **40**(2): p. 162–169.

26. Marshall, G.L., et al., *Dynamics of Financial Hardship in the United States: Health and Retirement Study 2006-2016.* J Gerontol Soc Work, 2022. **65**(3): p. 241–251.

27. Marshall, G.L. and R. Tucker-Seeley, *The association between hardship and self-rated health: does the choice of indicator matter?* Ann Epidemiol, 2018. **28**(7): p. 462–467.
